# Trade-offs among cost, integration, and segregation in the human connectome

**DOI:** 10.1162/netn_a_00291

**Published:** 2023-06-30

**Authors:** Junji Ma, Xitian Chen, Yue Gu, Liangfang Li, Cam-CAN, Ying Lin, Zhengjia Dai

**Affiliations:** Department of Psychology, Sun Yat-sen University, Guangzhou, China; Cambridge Centre for Ageing and Neuroscience (Cam-CAN), University of Cambridge and MRC Cognition and Brain Sciences Unit, Cambridge, United Kingdom; Guangdong Provincial Key Laboratory of Brain Function and Disease, Zhongshan School of Medicine, Sun Yat-sen University, Guangzhou, China

**Keywords:** Cost-efficiency trade-off, Segregation, Modularity, Diffusion MRI, Human brain network

## Abstract

The human brain structural network is thought to be shaped by the optimal trade-off between cost and efficiency. However, most studies on this problem have focused on only the trade-off between cost and global efficiency (i.e., integration) and have overlooked the efficiency of segregated processing (i.e., segregation), which is essential for specialized information processing. Direct evidence on how trade-offs among cost, integration, and segregation shape the human brain network remains lacking. Here, adopting local efficiency and modularity as segregation factors, we used a multiobjective evolutionary algorithm to investigate this problem. We defined three trade-off models, which represented trade-offs between cost and integration (Dual-factor model), and trade-offs among cost, integration, and segregation (local efficiency or modularity; Tri-factor model), respectively. Among these, synthetic networks with optimal trade-off among cost, integration, and modularity (Tri-factor model [*Q*]) showed the best performance. They had a high recovery rate of structural connections and optimal performance in most network features, especially in segregated processing capacity and network robustness. Morphospace of this trade-off model could further capture the variation of individual behavioral/demographic characteristics in a domain-specific manner. Overall, our results highlight the importance of modularity in the formation of the human brain structural network and provide new insights into the original cost-efficiency trade-off hypothesis.

## Author Summary

The human brain structural network is hypothesized to be organized under an optimal trade-off between cost and efficiency. However, the efficiency of segregated processing in this trade-off is overlooked. Adopting multiobjective evolutionary algorithm, we revealed that synthetic networks with optimal trade-off among cost, global efficiency, and modularity (Tri-factor model [*Q*]) could capture empirical brain network structure very well. Synthetic networks of Tri-factor model (*Q*) had a high recovery rate of structural connections and optimal performance in network features, especially in segregated processing capacity and network robustness. The morphospace of this model could further capture the variation of individual behavioral/demographic characteristics. These results highlight the indispensable role of modularity in shaping the human brain structural network and provide new insights into the original cost-efficiency trade-off hypothesis.

## INTRODUCTION

At the macroscale, the human connectome, characterized as a complex network with brain regions as nodes and white matter axonal pathways as connections, is the anatomical substrate of information communication and ultimately supports brain functions and cognition (Bullmore & Sporns, 2009, 2012; Sporns, Tononi, & Kötter, 2005). Identifying the principles that guide the wiring pattern of the human connectome promotes our understanding of human brain organization and how the organization evolves to support human brain functions. Recently, incorporating neuroimaging data, researchers found diverse factors that are related to the wiring of the human connectome, such as geometrical factors (e.g., Euclidean distance; Klyachko & Stevens, 2003; Markov et al., 2013; Roberts et al., 2016) and topological factors (e.g., matching index; Betzel et al., 2016). Although diverse network constraints were proposed, it remains an open question what fundamental and general principle underlies the various constraints on brain network configuration (Stiso & Bassett, 2018).

A review article proposed the hypothesis that the general principle of the human connectome wiring is the optimal trade-off between minimizing wiring cost and maximizing communication efficiency (Bullmore & Sporns, 2012). Wiring cost refers to the material cost that is needed in construction and maintenance of anatomical brain connections (Ahn, Jeong, & Kim, 2006; Mitchison, 1991), which is directly related to the length and density of connections in the brain network. With limited material resources, cost minimization places a strong constraint on brain network structure and leads to the emergence of several network features (e.g., distance-dependent connection pattern; Klyachko & Stevens, 2003; Markov et al., 2013; Samu, Seth, & Nowotny, 2014). However, only cost minimization is insufficient to explain the whole wiring pattern of the human connectome (Kaiser & Hilgetag, 2006). The existence of long-distance connections, hubs, and some other topological characteristics (e.g., small-worldness; Bassett & Bullmore, 2006, 2017; Liao, Vasilakos, & He, 2017; van den Heuvel & Sporns, 2013), which violates the cost minimization principle, has suggested the additional need to facilitate communication in the human brain network. Therefore, the human connectome was supposed to be constructed under the pressure of a cost-efficiency trade-off (Bullmore & Sporns, 2012). The influence of this trade-off was recently confirmed in the network of human (Ma et al., 2021) and *C. elegans*/macaque brains (Y. Chen et al., 2013, 2017). Notably, the above studies mainly focused on the trade-off between cost and global efficiency, which is the overall efficiency of information transfer among all pairs of brain regions in the network (Avena-Koenigsberger et al., 2014; Fornito et al., 2011). However, effective communication of human brain requires not only efficient global information integration, but also the capacity for segregated information processing (Sporns, 2013).

Segregation refers to specialized information processing that occurs within region clusters (e.g., modules or the neighbors of the node). The capacity of segregated information processing not only enables flexible and rapid reconfiguration of the brain network in response to different task demands, but also benefits network robustness (Wig, 2017; Sporns & Betzel, 2016). In the human macroscopic connectome, segregation can be described in two different ways: the efficiency of local cluster and modularity (Cohen & D’Esposito, 2016; Sporns, 2013). The efficiency of local cluster (i.e., local efficiency and clustering coefficient) measures the density of connections among neighboring brain regions, reflecting the efficiency of information transfer within regional subnetworks (Achard & Bullmore, 2007; Latora & Marchiori, 2001). Modularity characterizes network structure with dense intra-module connections and sparse inter-module connections (Meunier, Lambiotte, & Bullmore, 2010; Meunier et al., 2009). This arrangement of the human connectome allows efficient and mutually independent segregated processing within modules (Meunier et al., 2010). Moreover, the modular structure could give rise to richer distribution of information in the brain network and promote functional complexity (Gallos, Makse, & Sigman, 2012; Kaiser, Hilgetag, & Kötter, 2010). In sum, both segregation measures capture unique segregated properties that could not be simply explained by the trade-off between cost and global efficiency (Ma et al., 2021). Therefore, the cost-efficiency trade-off principle needs to be further extended by including segregation capacity. However, so far, direct evidence remains lacking for how segregation capacity participates in the cost-efficiency trade-off and how the trade-off among these factors (i.e., cost, global integration, and segregation) shapes the human connectome.

To address this issue, first, we modeled the wiring process driven by the trade-off among the factors of cost, integration, and segregation as multiobjective optimization problems, with the segregation factor expressed as local efficiency and modularity, respectively. For comparison, we also modeled the wiring process driven solely by the trade-off between cost and integration in the same way. Second, we implemented a multiobjective evolutionary algorithm (MOEA; Zhou et al., 2011) to solve the above problems. MOEA is one type of search algorithm for multiobjective optimization problems that consider two or more conflicting objectives. More specifically, MOEA imitates the natural evolutionary procedure of organisms to find a set of mutually non-dominated (equally good from the perspective of multiple objectives) solutions that can approach the Pareto front (composed of solutions with optimal but different trade-offs among objectives) of a multiobjective optimization problem (Tušar & Filipič, 2015; Zhou et al., 2011). Herein, MOEA was not for simulating the actual evolutionary processes of brain networks, but served as a phenomenological model that can generate synthetic networks yielding an optimal trade-off among constraints (e.g., cost, integration, and segregation). This approach has inherent advantages in solving multiobjective problems: (a) It has a good global searching ability that allows efficient exploration in high-dimensional solution space. (b) It can generate a set of solutions (i.e., synthetic networks in this study) that evenly approximate the entire Pareto front of the problem, which facilitates exploration of equivalently optimal but diverse trade-offs. Third, we compared the generated synthetic networks with empirical human brain networks in topological characteristics. We then determined the best trade-off model that could recover the most organization of empirical brain networks. Finally, considering the tight relationship between brain network organization and human behavioral/demographic characteristics (Cao et al., 2014; Heitger et al., 2012; Ingalhalikar et al., 2014; Park & Friston, 2013), we further examined whether the best trade-off model could also capture basic individual behavioral/demographic characteristics (i.e., age, gender, and fluid intelligence) through their variation of the brain network.

## MATERIALS AND METHODS

### Participants

In this study, to obtain empirical networks and corresponding demographic and behavioral scores, we used two independent datasets. The first dataset included 93 healthy college students (mean age = 18.95 ± 1.08 years old; 29 males) collected from the South China Normal University (SCNU dataset). All participants in this dataset did not have a history of neurological or psychiatric disorders, sensorimotor or cognitive impairment, or other anatomical injuries of the brain, and have provided informed consent before scanning. This study was approved by the Institutional Review Board in the Department of Psychology of Sun Yat-sen University. The second dataset was an openly available adult life span dataset collected from Cambridge Centre for Ageing and Neuroscience (Cam-CAN dataset; Shafto et al., 2014; Taylor et al., 2017). A sample of 589 participants (mean age = 54.01 ± 18.46 years old; 285 males) were acquired from this dataset. Participants were all healthy adults with normal or corrected-to-normal vision and hearing, scored 25 or higher on the Mini–Mental State Exam (MMSE), and had no history of drug or alcohol abuse, or of neurological disorders. All participants underwent a diverse set of neuropsychological tests, conducted cognitive tasks, and had MRI scans. Informed consent was obtained from all participants and the study was approved by the Cambridgeshire 2 Research Ethics Committee, United Kingdom. Notably, the SCNU dataset was used in the main analyses for exploring the optimal trade-off models (for details see Definitions of Trade-Off Models section). The Cam-CAN dataset was used for validating our main findings as an independent sample and examining behavioral relevance of the trade-off model.

### MRI Data Acquisition

For the SCNU dataset, all participants were scanned on a Siemens 3.0 Tesla MRI scanner (Siemens, Erlangen, Germany) at South China Normal University (Guangzhou, China). Headphones and foam pads were used to avoid interference of scanner noise and reduce head motion of participants during the scan. Participants were required to keep their eyes closed, stay awake without thinking about anything, and keep their heads fixed during the data acquisition. Structural T1-weighted images were collected using magnetization prepared by rapid gradient echo sequence: repetition time (TR) = 1,900 ms, echo time (TE) = 2.52 ms, flip angle = 9°, field of view (FOV) = 256 × 256 mm^2^, inversion time = 900 ms, matrix = 256 × 256, slices = 176, slice thickness = 1 mm, and voxel size = 1 × 1 × 1 mm^3^. The diffusion MRI (dMRI) data were collected using a single-shot spin echo / echo planar sequence with the following parameters: TR = 10,000 ms, TE = 90 ms, matrix = 128 × 128, FOV = 256 × 256 mm^2^, flip angle = 90°, and slice thickness = 2 mm without gap. The diffusion sensitizing gradients were applied along 64 noncollinear directions (b = 1,000 s/mm^2^), together with one acquisition without diffusion weighting (b = 0 s/mm^2^). Resting-state functional MRI data were also acquired, but the data were not used in this study.

For the Cam-CAN dataset, all participants were scanned on a 3T Siemens TIM Trio System at the MRC Cognition Brain and Sciences Unit, Cambridge, United Kingdom. Structural T1-weighted images were collected using magnetization prepared by rapid gradient echo sequence: repetition time (TR) = 2,250 ms, echo time (TE) = 2.99 ms, flip angle = 9°, field of view (FOV) = 256 × 240 mm^2^, inversion time = 900 ms, slices = 192, slice thickness = 1 mm, and voxel size = 1 × 1 × 1 mm^3^. The dMRI images were acquired with a twice-refocused spin echo sequence: TR = 9,100 ms, TE = 104 ms, matrix = 128 × 128, FOV = 192 × 192 mm^2^, slice = 66, and voxel size = 2 × 2 × 2 mm^3^.

### Image Preprocessing and Anatomical Brain Network Construction

Preprocessing and network construction procedures were identically applied on imaging data of two datasets. Specifically, all the dMRI images were preprocessed using the standard preprocessing procedure of the PANDA toolbox (Cui et al., 2013). The preprocessing procedure included brain mask estimation, skull-stripping, eddy current, head motion correction, and diffusion tensor metrics calculation (i.e., voxel-wise tensor matrix and fractional anisotropy [FA]). Furthermore, the T1-weighted images were aligned to the AC-PC line and then segmented using SPM8 software to obtain the white matter (WM) binary mask (with WM probability threshold > 0) in the T1 native space. Finally, the WM mask was transformed into the native diffusion space of each participant for subsequent WM tractography with the inverse transformation matrix, which was estimated in coregistration of the FA image to T1.

Then we defined nodes and edges of the human macroscopic connectome. For nodes, we used the Automated Anatomical Labeling (AAL) atlas (Tzourio-Mazoyer et al., 2002) to define 90 nodes covering the whole brain. Specifically, an inverse warping transformation from the standard MNI space to the native diffusion space can be obtained based on coregistering the individual FA image to T1-weighted image and then nonlinearly registering to the ICBM152 template. The AAL was then inversely warped back to individual native diffusion space by applying this inverse transformation to define the network nodes. For edges, we used deterministic tractography to construct the network. All possible streamlines were reconstructed by seeding from the voxel within the WM mask. A streamline was started from a seed, which was distributed at the center of each voxel with an FA value greater than 0.2. The streamline was terminated when it reached a voxel with a turning angle greater than 45° or an FA value less than 0.2, or out of the WM mask. The edge of each pair of nodes was defined as one when existing at least one streamline with two endpoints located in the corresponding node areas of the AAL atlas (Collin et al., 2014; Fukushima et al., 2018; van den Heuvel & Sporns, 2011; Zalesky et al., 2016). Therefore, we constructed a 90 × 90 binary structural network for each participant. We then constructed a group-level structural brain network by retaining edges that existed in more than 50% of the participants’ structural brain networks (Fukushima et al., 2018; Roberts et al., 2017; Zalesky et al., 2016). This group threshold is suggested to have a good balance between controlling false positive and false negative rates of the constructed connections (de Reus & van den Heuvel, 2013).

### Definitions of Trade-Off Models

**Figure 1.**
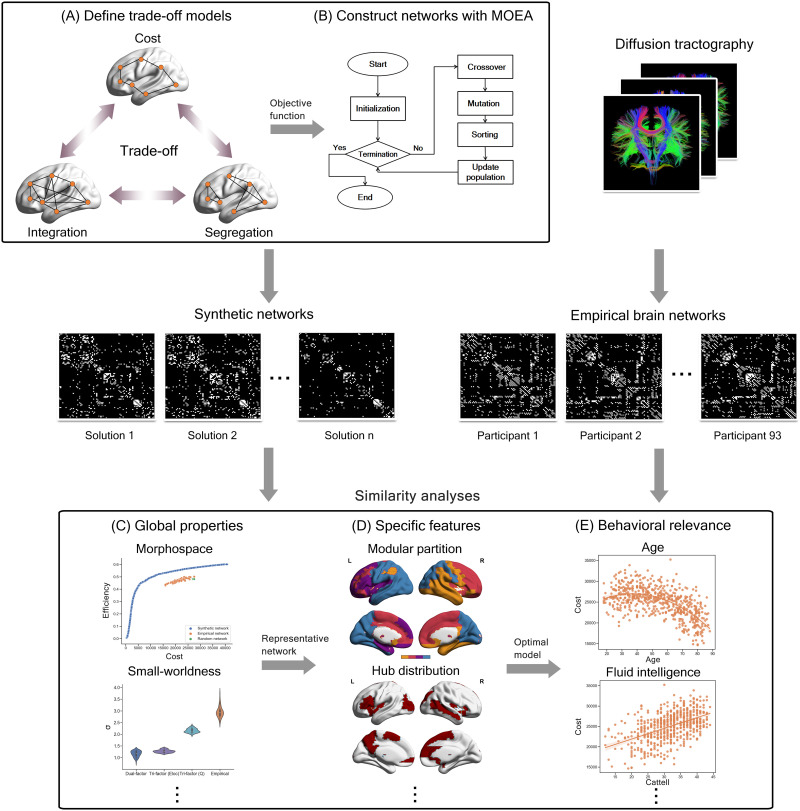
Schematic diagram of study design. (A) Model the trade-off principles (e.g., trade-off among cost, integration, and segregation) as multiobjective problems. (B) Implement MOEA (multiobjective evolutionary algorithm) pipeline and construct synthetic networks under corresponding optimal trade-offs. (C) Analyze the similarity between synthetic networks and empirical brain networks in the aspect of global properties (e.g., morphospace and small-worldness). (D) Analyze the similarity between synthetic networks and empirical brain networks in the aspect of more specific features (e.g., modular partition and hub distribution). (E) Investigate the behavioral relevance of optimal trade-off model obtained from previous steps.

To systematically examine how different trade-off models shape the human connectome, we needed to construct synthetic networks under specific trade-off models and then compare them with empirical brain networks (Figure 1). Three models were defined to simulate the wiring process driven by optimizing the trade-offs among the above factors. In particular, since wiring cost is related to both the number and the length of connections (Ahn et al., 2006; Mitchison, 1991), the cost factor here was formulated as the sum of Euclidian distances between centroids of connected regions (Y. Chen et al., 2013, 2017; Ma et al., 2021), rather than measures that simply consider connection numbers (e.g., degree); the integration factor was formulated as the global efficiency (*Eg*) of the network; and the segregation factor was formulated either as the local efficiency (*Eloc*) of network or as the modularity (*Q*) of the network. The network-level local efficiency was computed as the average of nodal local efficiency of all nodes. For detailed mathematical definitions of the above measures, refer to the Supporting Information. Based on the three factors, each trade-off model defined two optimization objectives to capture the competing relationship between wiring cost and communication efficiency. The cost objective *F*_c_ was defined directly based on the cost factor and was set the same in all the models, while the efficiency objective *F*_e_ was defined differently to express sole concern on network integration or hybrid concern on network integration and segregation in different forms. To unify the optimization direction with the cost objective (i.e., minimization), the definitions of *F*_e_ were all formulated in a way that smaller values indicated better efficiency. In detail, the definitions of *F*_e_ in the three models are summarized below.

- Dual-factor model: cost and integration
  $$F_{e}=1-Eg$$
- Tri-factor model (*Eloc*): cost, integration, and segregation (local efficiency)
  $$F_{e}=w_{\text{Eloc}}\left(1-Eg\right)+\left(1-w_{\text{Eloc}}\right)\left(1-\text{Eloc}\right)$$
- Tri-factor model (*Q*): cost, integration, and segregation (modularity)
  $$F_{e}=w_{Q}\left(1-Eg\right)+\left(1-w_{Q}\right)\left(1-Q\right)$$

In the above definitions, *w*_*Eloc*_ and *w*_*Q*_ were both weights in the range of (0, 1) for specifying the relevant importance imposed on network segregation capacity when it was simultaneously optimized with integration capacity. Parameter sensitivity investigations showed that the two Tri-factor models performed the best in recovering the empirical brain connectome when *w*_*Eloc*_ = 0.9 and *w*_*Q*_ = 0.8 (Supporting Information Figure S1). These settings were thus adopted in subsequent analyses. Notably, the Dual-factor model used in the current study was highly consistent with the one proposed in our previous work (Ma et al., 2021), except for the trivial difference in the mathematical formulation of *F*_*e*_ (the former and the current formulations achieved a correlation of 0.983 [*p* < 0.001] on the SCNU sample). The use of current formulation was for consistent comparisons across the trade-off models.

Moreover, we further defined an efficiency index, computed as 1 − *F*_*e*_, for each model. The higher value of this index suggests better communication efficiency of the network. Taking this index and the *F*_*c*_ objective as axes, we could construct a morphospace for each model, respectively. Location of networks in the morphospace reflects their performance in objective functions and their relative trade-off between objectives under the corresponding trade-off models, which allows comprehensive comparisons between networks.

### Construction of Synthetic Networks Using a Multiobjective Evolutionary Algorithm

Based on the above three models that represented different trade-offs underpinning the human connectome organization, we tried to generate synthetic networks that achieved the optimal trade-offs between the objectives of each model, respectively. Here, we applied the non-dominated sorting genetic algorithm (NSGA-II; Deb et al., 2002; https://www.egr.msu.edu/~kdeb/codes.shtml), a popular multiobjective evolutionary algorithm (MOEA), to construct synthetic networks. Following the procedure in our previous study (Ma et al., 2021), the implementation of the NSGA-II algorithms (Deb et al., 2002) was described as follows:

Step 1: Population initialization. The algorithm started with initialization of a population of synthetic networks, which were binary vectors representing upper triangle of 90 × 90 adjacent matrices (i.e., candidate solutions to trade-off models). Individual (i.e., synthetic network) in the population was initialized by randomly assigning a value (one or zero) to each entry, which indicates whether each node pair was connected. The probability of assigning one to entries is 0.1. According to the problem dimension (i.e., the number of decision variables to be optimized), the population size was set to 200.

Step 2: Reproduction of the population. Imitating the reproduction in natural evolutionary process, MOEAs design crossover operators to preserve high-quality genes (i.e., values of decision variables) for subsequent generations. Specifically, the operator randomly chose a portion of edge entries and swapped between synthetic networks to generate offspring networks. Two hundred offspring networks, forming an offspring population, were generated at the current step.

Step 3: Mutation of the population. Like mutation of chromosomes in nature, mutation in MOEAs is a low-probability but indispensable step, which can introduce diversity into the population and thereby enhance global search ability in the solution space. Here, the mutation operator randomly flipped the edge entries (i.e., 1 to 0 or vice versa) in the network with a low probability (probability = 0.1). The 200 mutated networks were then added to the offspring population.

Step 4: Selection of the population. To select elites for the next generation, the selection operator applied the fast non-dominated sorting approach (Deb et al., 2002) to rank the candidate networks according to their fitness (i.e., mutual dominance relationship of objective values) and local diversity (i.e., crowding distance between solutions in the objective space). For the individuals in the union of the current population and its offspring population, only the 200 that survived the selection process would enter the population of the next generation.

Step 5: Termination check. The algorithm terminated when one of the following conditions was satisfied: the population stopped evolving (i.e., < 5% of individuals were changed or the difference of mean objective values < 0.1%) for 20 consecutive generations, or the number of generations reach 2,000. Otherwise, the algorithm returned to Step 2 and started a new generation based on the updated population of the networks derived from Step 4. Evaluation of objective functions was performed right after new synthetic networks were generated at each step.

Considering that MOEA is a probabilistic algorithm and its results can vary across different runs, we ran the algorithm 30 independent times. The resulting solutions (i.e., synthetic networks) from all the runs were then sorted by the fast non-dominated sorting approach (Deb et al., 2002) to select the set of solutions that approximated the Pareto front (Tušar & Filipič, 2015; Zhou et al., 2011) of the problem (i.e., the best trade-offs of each model) for subsequent analyses. It should be noted that our MOEA approach is a theory-driven method. Instead of generating a brain-like network, MOEA only generates networks that optimize trade-off among specific objectives (e.g., cost-efficiency trade-off; Bullmore & Sporns, 2012). This optimization process did not require guiding information from empirical brain networks. Hence, including more constraints does not necessarily make the resulting synthetic networks more similar to the empirical ones.

### Recovery Rate of Synthetic Networks

Using the above NSGA-II algorithm, for each of the three models, a population of synthetic networks that approximated the optimal trade-off between the cost and efficiency objectives was generated. To explore the trade-off models underlying the human brain, we first compared their capacity in recovering connections of the group-level empirical brain structural networks. This capacity was evaluated by the recovery rate *R*, which measures the ratio of overlapping entries between the adjacency matrices of synthetic and empirical networks (Y. Chen et al., 2013, 2017; Costa, Kaiser, & Hilgetag, 2007; Ma et al., 2021). That is,

$$R=\sqrt{R_{0}R_{1}},$$

where *R*_0_ and *R*_1_ are the recovery rates regarding 0 and 1 entries in the adjacency matrices (diagonal entries omitted), respectively. To examine how connections between regions with different Euclidian distance were recovered under the trade-off models, we also calculated the recovery rates within different distance groups (i.e., 0–20 mm, 20–40 mm, 40–60 mm, 60–80 mm, 80–100 mm, and 100–120 mm).

Besides the recovery rate of trade-off models, we also computed the recovery rate from random networks to empirical networks as benchmarks for comparison. Specifically, we constructed topological random networks (Maslov & Sneppen, 2002) that connections were randomly rewired while preserving the numbers of nodes and edges, and the degree distribution of the empirical brain network. One hundred random networks of the random model were generated and their recovery rates were computed, respectively.

### Topological Characteristics of the Synthetic Networks

Further, we would like to examine how well the synthetic networks of different models capture the topological features of empirical brain networks. Here, we computed network-level topological measures that were previously suggested to be important features of the human connectome (Bullmore & Sporns, 2009; Rubinov & Sporns, 2010) and related to cost and efficiency (i.e., integration and segregation; Liao et al., 2017; Sporns & Betzel, 2016; Samu et al., 2014). The selected metrics include the small-world metrics (i.e., clustering coefficient [Cp], characteristic path length [Lp], normalized clustering coefficient [*γ*], normalized characteristic path length [*λ*], and small-worldness [(*σ*]), efficiency metrics (i.e., global efficiency [*Eg*] and local efficiency [*Eloc*]), and modularity metrics (i.e., modularity [*Q*] and the number of modules [Mn]). These metrics describe the network integrated and segregated processing capacity from different angles. Lower characteristic path length and normalized characteristic path length and higher global efficiency of network all reflect higher global processing capacity. Higher scores in clustering coefficient, normalized clustering coefficient, local efficiency, and modularity are associated with better segregated processing capacity. Small-worldness reflects a balance between network integration and segregation. Among these metrics, global efficiency was trained in all the trade-off models, local efficiency was trained in the Tri-factor model (*Eloc*), and modularity was trained in the Tri-factor model (*Q*). The rest of the measures were not directly trained in any trade-off models. Detailed equations and definitions of the topological measures are presented in the Supporting Information. Calculation of the above topological metrics was performed using the Graph Theoretical Network Analysis Toolbox (GRETNA; J. Wang et al., 2015) and Brain Connectivity Toolbox (BCT; Rubinov & Sporns, 2010). Notably, to avoid the potential effect of cost on these metrics, only the synthetic networks whose cost objective values were distributed in the cost range of the empirical networks at the individual level were selected for comparison.

To measure the overall ability of trade-off models to recover the nine topological characteristics above, we developed a distance-based cost function, termed topological dissimilarity. Specifically, first, we normalized each topological metric across all networks, including synthetic networks of the three models and empirical brain networks, with z-score normalization. Then we computed the centroid of empirical brain networks by averaging each normalized metric score across empirical brain networks. For each synthetic network, the topological dissimilarity was defined as the Euclidean distance between normalized metric scores of the synthetic network and normalized metric scores of the empirical centroid.

### Specific Features of the Representative Synthetic Network

Besides the above network-level features, some other properties at a more specific level (e.g., nodal degree centrality) were also crucial for the network function (e.g., integration) of the human brain (Aerts et al., 2016; Sporns, 2013; van den Heuvel & Sporns, 2013). To investigate how these specific features emerge under the pressure of different trade-offs, we extracted one representative network from the approximated Pareto sets of the three trade-off models, respectively, and examined their relationship with the group-level empirical brain network. The representative network of each trade-off model was defined as the synthetic network that had the highest recovery rate within the cost range of the SCNU sample, which ensured the extracted representative network would be comparable with the group-level empirical network. The similarity between representative networks and the group-level empirical brain network was examined in three aspects: modular structure, nodal degree centrality, and robustness of network. The modular structure of networks was compared by their similarity in modular partition obtained from the Louvain community detection algorithm (Blondel et al., 2008). The degree centrality was compared through the correlation of nodal degree centrality and the overlap of hub distribution between synthetic networks and empirical brain network. As for robustness, we examined the degree of network degeneration under computational attacks (i.e., random attack and targeted attack; Crossley et al., 2014; Kaiser, Martin, Andras, & Young, 2007) in terms of global and local efficiency. Notably, all the above specific metrics were not trained in any trade-off models.

### Behavioral Relevance of the Optimal Trade-Off Model

After the above analyses, we could infer the optimal trade-off model that best reproduced features of empirical human brain networks. Considering the tight relationship between the brain connectome and individual characteristics, we were interested in whether the optimal trade-off model could also capture the basic demographic and behavioral characteristics of individuals. Analyses of the current section were performed on the Cam-CAN sample, as it has a relatively large sample size and considerable individual variance in demographic/behavioral scores (Shafto et al., 2014; Taylor et al., 2017). Through computing the objective values (i.e., *F*_*c*_ and 1 − *F*_*e*_) of networks, we mapped the structural networks of Cam-CAN participants to the morphospace of the optimal trade-off model. Three indices were derived to characterize the spatial location of each participant to reflect features of his/her structural brain network. The three indices were the two axis values (i.e., *F*_c_ and 1 − *F*_e_) and the slope of the vector representing individual network (i.e., [1 − *F*_e_]/*F*_c_), which indicates the relative trade-off between the wiring cost and the communication efficiency. The relationship between these indices and demographic/behavioral data of participants were then investigated. For demographic data, we examined the relationship between age and the three indices, which was examined through regression analysis, and the gender difference in each index (age controlled). For behavioral data, we extracted the total score of the Cattell test (Cattell, 1963), which measures the fluid intelligence of participants. Of the 589 participants in the Cam-CAN sample, 575 participants have finished the Cattell test. Pearson correlation analyses were performed between the morphospace indices and behavioral scores on the corresponding subsample of participants.

To examine the significance of results in above analyses, we performed permutation tests. First, we shuffle the demographic or behavioral values across participants. Then we computed the correlation value (e.g., Pearson correlation coefficients) between shuffled valued and morphospace indices, or the sexual difference based on shuffled gender groups. These operations were then repeated 10,000 times to obtain a null distribution of corresponding analysis. The ratio of values in the null distribution that is higher than actual correlation or difference value will be defined as the significance of corresponding results.

## RESULTS

### Synthetic Networks and Morphospace of Trade-Off Models

**Figure 2.**
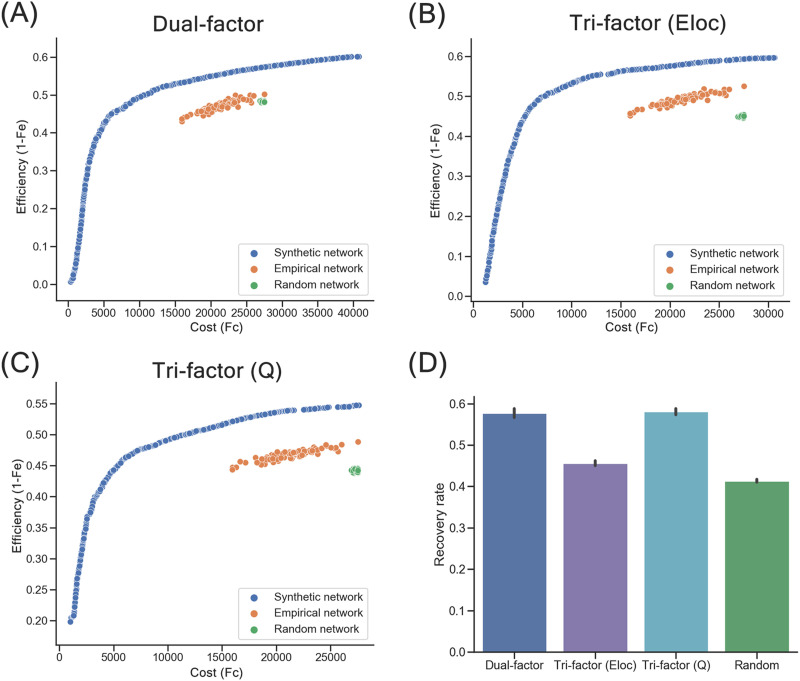
Synthetic networks of trade-off models. (A) Distribution of synthetic networks (blue points), empirical brain networks (orange points), and random networks (green points) in morphospace of the Dual-factor model. (B) Distribution of networks in morphospace of the Tri-factor model (*Eloc*). (C) Distribution of networks in morphospace of the Tri-factor model (*Q*). Notably, the values of the *F*_e_ objective were transformed by 1 − *F*_e_ (i.e., efficiency index). (D) Recovery rates of synthetic networks and random networks. Recovery rates were significantly different between network groups (*p*s < 0.001), except for comparison between Dual-factor model and Tri-factor model (*Q*) (*p* = 0.608).

Using the proposed MOEA, three final sets of synthetic networks achieving optimal trade-off of the corresponding models were generated (Dual-factor: 347 networks [connection number: 337.133 ± 250.497; density: 0.084 ± 0.063]; Tri-factor [*Eloc*]: 312 networks [connection number: 227.398 ± 149.187; density: 0.057 ± 0.037]; Tri-factor [*Q*]: 281 networks [connection number: 252.645 ± 153.270; density: 0.063 ± 0.038]). These networks (blue points in Figure 2A–C) constituted the approximated Pareto fronts that represented a diverse and optimal trade-off in their own morphospace, where efficiency of networks was computed based on the definition in the corresponding trade-off model. Compared with the synthetic networks, the 93 individual empirical brain networks (connection number: 477.688 ± 33.875; density: 0.119 ± 0.009; orange points) were distributed along a similar direction as the optimal fronts (i.e., fronts composed of synthetic networks) within a narrower range, that is, as the networks’ efficiency gets higher their cost also increases to a similar degree, suggesting a similar trade-off in the synthetic and empirical network groups. However, these individual empirical brain networks were all dominated by the synthetic networks and mainly distributed at the top right area of the morphospace, which means empirical brain networks prioritize efficiency more in the trade-offs but less optimal trade-offs compared with the synthetic networks of the three models. Random networks (green points) were also distributed at the suboptimal area of the space, all dominated by empirical brain networks. To reveal how synthetic networks of different models related to each other in the morphospace, we further evaluated the synthetic networks using the efficiency objective functions of the three trade-off models and then mapped the results onto the corresponding morphospace. We observed that for each model, the generated synthetic networks dominated those from the other two models in the morphospace of the model, especially within the cost range of empirical brain networks (Supporting Information Figure S5), suggesting the unique optimality of the synthetic networks under their own models.

In terms of the recovery rate that quantitatively measures how well the connections of group-level empirical brain networks were recovered, significant differences were found among synthetic network groups and random networks (one-way analysis of variance [ANOVA]: *F* = 605.140, *p* < 0.001). All three trade-off models showed significantly higher recovery rates than random networks (*R* = 0.414 ± 0.016; two-sample *t* test: *t*s ≥ 13.547, *p*s < 0.001; Figure 2D). Among these trade-off models, the Tri-factor model (*Q*) (*R* = 0.581 ± 0.030) and Dual-factor model (*R* = 0.577 ± 0.049) had the highest recovery rates, which were significantly higher than the Tri-factor model (*Eloc*) (*R* = 0.456 ± 0.024; two-sample *t* test: *t*s ≥ 18.377, *p*s < 0.001). No significant difference was observed between the Tri-factor model (*Q*) and the Dual-factor model (two-sample *t* test: *t* = 0.514, *p* = 0.608). Note that to avoid the potential effect of cost, the recovery rate analysis and the global topological analyses below focused on only synthetic networks whose cost values fell in the range of individual empirical brain networks (*F*_*e*_ range: 15,958–27,544), resulting in 77 networks of the Dual-factor model (connection number: 465.013 ± 57.365; density: 0.116 ± 0.018), 65 networks of the Tri-factor model (*Eloc*) (connection number: 437.013 ± 57.796; density: 0.109 ± 0.014), and 70 networks of the Tri-factor model (*Q*) (connection number: 460.532 ± 55.125; density: 0.115 ± 0.014).

### Global Properties Under Trade-Off Models

**Figure 3.**
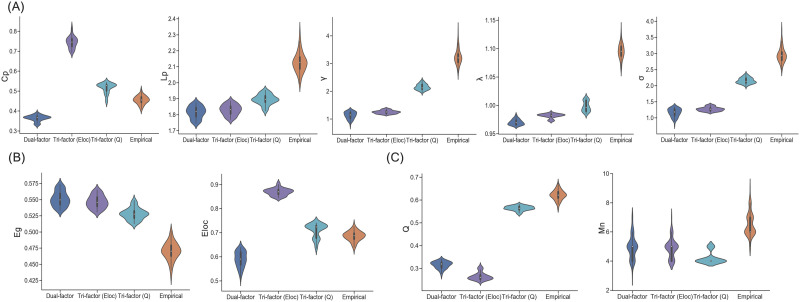
Network-level topological characteristics of networks. (A) Clustering coefficient (Cp), characteristic path length (Lp), normalized clustering coefficient (*γ*), normalized characteristic path length (*λ*), and small-worldness (*σ*) of synthetic networks and empirical brain networks. All the small-world metrics were significantly different between groups (*p*s < 0.05). (B) Global efficiency (Eg) and local efficiency (El) of synthetic networks and empirical brain networks. All the small-world metrics were significantly different between groups (*p*s < 0.05). (C) Modularity (*Q*) and module number (Mn) of synthetic networks and empirical brain networks. Modular metrics were significantly different between network groups (*p*s < 0.001), except for comparison between module number of Dual-factor model and Tri-factor model (*Eloc*) (*p* = 0.718). Note, the synthetic networks are the networks whose cost values are distributed in the range of the SCNU dataset.

Synthetic networks derived from the three different trade-off models also capture network-level topological features of empirical brain networks to varying degrees. For the small-world-related metrics (Figure 3A), similar to empirical brain networks, synthetic networks of all models showed a small-world structure (mean *σ* > 1; one-sample *t* test: *t*s ≥ 10.863, *p*s < 0.001). However, significant differences of small-world metrics were also observed among network groups (one-way ANOVA: *F*s ≥ 881.836, *p*s < 0.001). The Tri-factor model (*Q*) achieved the best performance in small-worldness among the trade-off models (two-sample *t* test: *t*s ≥ 53.324, *p*s < 0.001), but relatively lower than individual empirical brain networks (two-sample *t* test: *t* = −26.710, *p* < 0.001). More specifically, compared with the Dual-factor model proposed in our previous study (Ma et al., 2021), the Tri-factor model (*Q*) provided significant improvement in (normalized) clustering coefficient (two-sample *t* test: *t*s ≥ 44.180, *p*s < 0.001) with the price of longer (normalized) characteristic path length (two-sample *t* test: *t*s ≥ 13.710, *p*s < 0.001). The Tri-factor model (*Eloc*) also brought a small improvement on the small-world metrics (i.e., [normalized] clustering coefficient and small-worldness), but not as much as the Tri-factor model (*Q*) did (two-sample *t* test: *t*s ≥ 6.367, *p*s < 0.001).

For efficiency metrics (Figure 3B), similar patterns of group difference were also observed (one-way ANOVA: *F*s ≥ 903.750, *p*s < 0.001). Compared with the Dual-factor model, synthetic networks of the Tri-factor model (*Q*) sacrificed performance in global efficiency (two-sample *t* test: *t* = −13.533, *p* < 0.001) for an obvious improvement in local efficiency (two-sample *t* test: *t* = 23.215, *p* < 0.001). Both efficiency metrics were significantly higher in the Tri-factor model (*Q*) than individual empirical brain networks (two-sample *t* test: *t*s > 5.860, *p* < 0.001). The Tri-factor model (*Eloc*) showed a similar pattern as the Tri-factor model (*Q*).

For modularity metrics (Figure 3C), these network groups again exhibited significant differences with each other (one-way ANOVA: *F*s ≥ 168.520, *p*s < 0.001). Across the trade-off models, synthetic networks of the Tri-factor model (*Q*) had the highest modularity (two-sample *t* test: *t*s ≥ 111.375, *p*s < 0.001), but relatively lower than individual empirical brain networks (two-sample *t* test: *t* = −20.289, *p* < 0.001). Moreover, the modular number of the Tri-factor model (*Q*) was lower than other networks (two-sample *t* test: *t*s ≤ −6.870, *p*s < 0.001). Detailed descriptive data of the above topological metrics are presented in Table 1. Besides the synthetic networks and empirical brain networks, we also computed topological metrics on 100 topological random networks (Maslov & Sneppen, 2002) as the benchmark.

**Table 1.** Network-level graph metrics. Cp, clustering coefficient; Lp, characteristic shortest path length; *γ*, normalized clustering coefficient; *λ*, normalized characteristic shortest path length; *σ*, small-worldness; *Eg*, global efficiency; *Eloc*, local efficiency; *Q*, modularity; Mn, module number; Value, mean ± *SD* of the metric score; *t*, *t* scores of two-sample *t* test between metric scores of corresponding network group and random networks. Since computation of *γ*, *λ*, and *σ* have been normalized with random networks, we do not provide *t* scores for these metrics. *** *p* < 0.001, ^n.s.^ *p* > 0.05.

Graph metrics	Dual-factor		Tri-factor (*Eloc*)		Tri-factor (*Q*)		Empirical	
	Value	*t*	Value	*t*	Value	*t*	Value	*t*
Cp	0.364 ± 0.015	135.632***	0.745 ± 0.029	200.035***	0.516 ± 0.026	144.645***	0.455 ± 0.021	148.507***
Lp	1.815 ± 0.038	−67.557***	1.830 ± 0.033	−73.119***	1.895 ± 0.032	−54.702***	2.128 ± 0.063	8.364***
*γ*	1.127 ± 0.130	–	1.250 ± 0.059	–	2.155 ± 0.106	–	3.241 ± 0.287	–
*λ*	0.970 ± 0.005	–	0.982 ± 0.004	–	0.998 ± 0.009	–	1.095 ± 0.014	–
*σ*	1.161 ± 0.130	–	1.274 ± 0.064	–	2.158 ± 0.091	–	2.957 ± 0.237	–
*Eg*	0.551 ± 0.012	59.312***	0.547 ± 0.010	65.020***	0.528 ± 0.009	50.142***	0.470 ± 0.014	−8.379***
*Eloc*	0.590 ± 0.032	115.799***	0.869 ± 0.015	280.944***	0.709 ± 0.030	154.996***	0.686 ± 0.021	196.516***
*Q*	0.315 ± 0.016	14.946***	0.265 ± 0.017	−10.748***	0.564 ± 0.010	188.129***	0.621 ± 0.022	141.171***
Mn	4.922 ± 0.791	−13.799***	4.877 ± 0.673	−14.277***	4.200 ± 0.403	−23.080***	6.516 ± 0.789	−0.560^n.s.^

To access the ability of trade-off models to capture the above topological properties, we further computed a cost function (i.e., topological dissimilarity) for each model. Results demonstrate that the Tri-factor model (*Q*) had the statistically lowest topological dissimilarity among the three models (two-sample *t* test: *t*s ≤ −51.609, *p*s < 0.001; Supporting Information Figure S4A). Overall, the Tri-factor model (*Q*) revealed relatively better performance in capturing both integrated (e.g., global efficiency) and segregated (e.g., modularity) topological properties of empirical brain networks, regardless of whether these properties were trained.

### Representative Synthetic Network and Pattern of Distance-Dependent Connections

**Figure 4.**
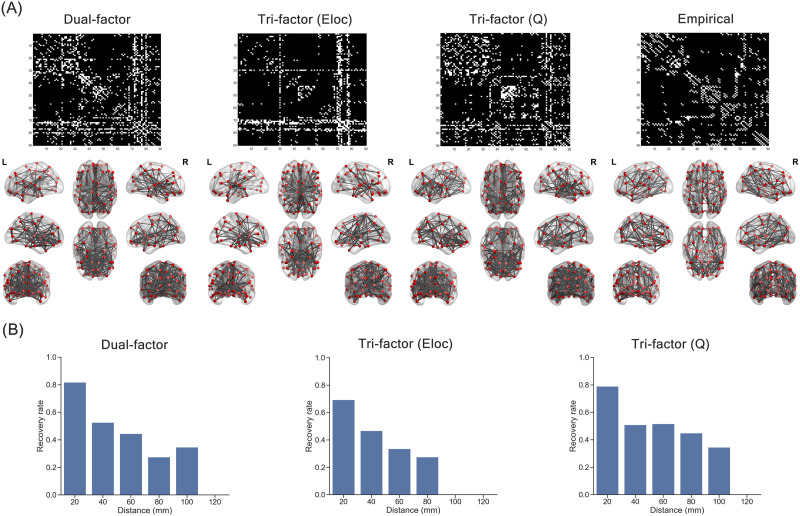
Representative synthetic network. (A) Adjacency matrices (upper panel) and brain maps (lower panel) of the representative synthetic networks and the group-level empirical brain network. The white color in the adjacency matrices indicates the existence of edges. (B) The recovery rate of edges with different distances.

As mentioned in the Materials and Methods section, to further examine more specific features of networks, we extracted a representative synthetic network from each trade-off model. Figure 4A presents the adjacency matrices and the brain maps of the three representative synthetic networks and the group-level empirical brain network (connection number: 388; density: 0.097). From visual inspection, the representative networks of the Dual-factor model (connection number: 591; density: 0.148) and the Tri-factor model (*Q*) (connection number: 576; density: 0.144) were more similar to the group-level empirical brain network. The representative network of the Tri-factor model (*Eloc*) (connection number: 429; density: 0.107) was relatively sparser, with connections mainly concentrating on a small group of regions (i.e., global hubs). Consistent with visual inspection, the overall recovery rates of the representative synthetic network from the Dual-factor model (*R* = 0.636) and the Tri-factor model (*Q*) (*R* = 0.632) were relatively high compared with the Tri-factor model (*Eloc*) (*R* = 0.503).

We then divided the connections in each representative synthetic network into six bins according to the Euclidean distance between region pairs (i.e., 0–20 mm, 20–40 mm, 40–60 mm, 60–80 mm, 80–100 mm, and 100–120 mm) and found that recovery of connections was not uniform. Recovery rates of all three models shared a pattern that short-distance connections (Euclidian distance < 40 mm) were better recovered (0.467 < *Rs* < 0.817) than middle- or long-distance connections (Euclidian distance ≥ 40 mm, *Rs* < 0.516) in the synthetic networks (Figure 4B). To quantify the ability of recovering middle- or long-distance connections, we computed the area under curve (AUC) of the bars at middle- or long-distance bins (i.e., Euclidian distance ≥ 40 mm; Figure 4B). Compared with the Dual-factor model (AUC = 13.421) and the Tri-factor model (*Eloc*) (AUC = 8.856), the representative network of the Tri-factor model (*Q*) could recover relatively more middle- or long-distance connections. In general, these findings suggested that local connections are preferable in all trade-off models and the Tri-factor model (*Q*) was able to capture more middle- or long-distance connections than others.

### Modular Structure of Synthetic Networks Under Trade-Off Models

**Figure 5.**
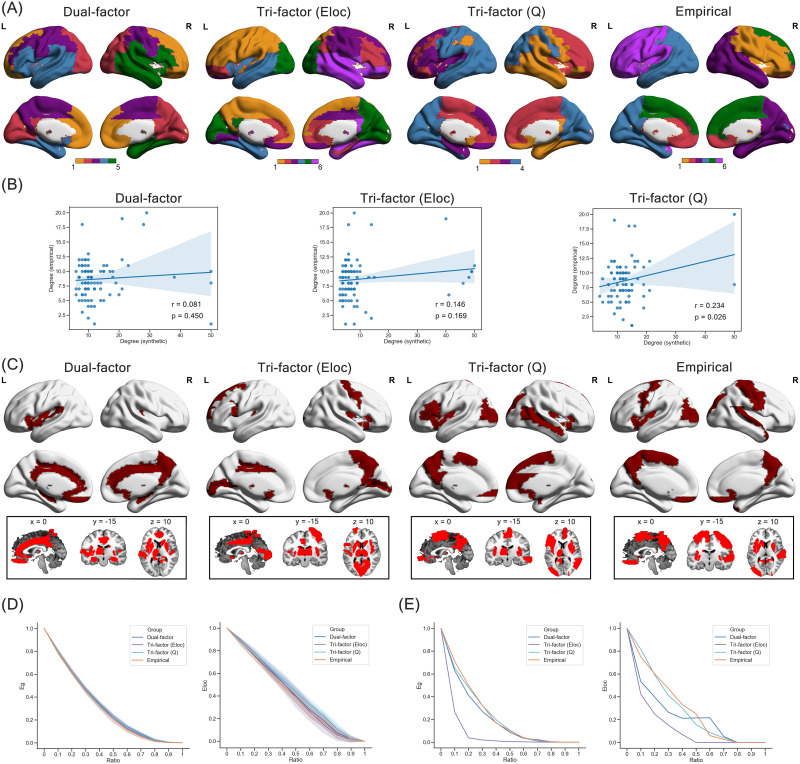
Network features of representative synthetic network. (A) Modular partition of synthetic networks and empirical brain network. (B) Scatterplot of degree centrality between synthetic networks and empirical brain network. (C) Spatial distribution of hub regions (red areas) in the synthetic network and empirical brain network. (D) Degeneration of global efficiency (Eg) and local efficiency (El) under random attacks on the synthetic and empirical brain networks. “Ratio” indicates the ratio of deleted nodes in the current step, and the shadow of lines indicates the range of 1 standard deviation. (E) Degeneration of global efficiency (Eg) and local efficiency (El) under targeted attacks on the synthetic and empirical brain network. “Ratio” indicates the ratio of deleted nodes in the current step.

The above findings of global modularity metrics suggested the existence of modular structure in both synthetic networks and individual empirical brain networks. To further examine similarity among their modular structure, we considered the modular partition of the representative synthetic networks and the group-level empirical brain network (Figure 5A). Consistent with the above patterns of global properties, the modularity score was higher in the group-level empirical brain network (modularity score = 0.528) and the synthetic network of the Tri-factor model (*Q*) (modularity score = 0.538), but lower in the synthetic networks of the Dual-factor model (modularity score = 0.321) and the Tri-factor model (El) (modularity score = 0.287), suggesting that the former two networks possessed stronger modular structure. In addition, the modular partition of the Tri-factor model (*Q*) was relatively more similar to that of the group-level empirical brain network (Kappa index = 0.413) than that of the Dual-factor model (Kappa index = 0.370) and the Tri-factor model (El) (Kappa index = 0.312). Therefore, the synthetic network of the Tri-factor model (*Q*) not only exhibited a stronger modular structure but also reproduced a more similar modular structure as the group-level empirical brain network.

### Nodal Degree Centrality and Spatial Distribution of Hub Regions

The second type of specific features we investigated was the nodal degree centrality (Figure 5B). Correlation results revealed that the nodal degree centrality of both the Dual-factor model (*r* = 0.081, *p* = 0.450) and the Tri-factor model (El) (*r* = 0.146, *p* = 0.169) was not significantly correlated with that in the group-level empirical brain network. Only in the Tri-factor model (*Q*) did we find a significant positive correlation (*r* = 0.234, *p* = 0.026). Then we identified hub regions in each network by selecting regions with the top 20% degree centrality. The spatial pattern of hub regions is shown in Figure 5C. We could observe that hub regions of the dual-factor model mainly concentrated at the subcortical area of the brain (e.g., thalamus), whilet hub regions of the two Tri-factor models distributed more dispersedly around the whole brain. To quantify the similarity of the hub distribution, we evaluated the number of overlapping hub regions between synthetic and group-level empirical brain networks. We found that the Tri-factor model (*Q*) (seven overlapping hubs; e.g., left insula, left precuneus) had more overlapping hub regions than the Dual-factor model (six overlapping hubs) and the Tri-factor model (*Eloc*) (five overlapping hubs). Together, our results suggested that the connection pattern of the Tri-factor model (*Q*) could partially capture the relative importance of brain regions in the group-level empirical brain network, which could not be achieved by the other trade-off models.

### Robustness of Synthetic Networks Under Trade-Off Models

Another important property we investigated was the robustness of network. Under random attacks (Figure 5D), we found that the robustness was different among synthetic networks and the group-level empirical brain network at most steps (one-way ANOVA: *F*s > 4.048, *p*s < 0.008), except for the steps of removing 10% (for preserved global efficiency) and 90% of nodes (for preserved local efficiency). The preserved network functions (i.e., global efficiency and local efficiency) of the synthetic networks at most steps were slightly better than the group-level empirical brain network. Specifically, the Dual-factor model showed better preserved global efficiency at steps from 0.2 to 0.9 (two-sample *t* test: *t*s > 3.722, *p*s < 0.001), and better preserved local efficiency at steps of 0.3 to 0.4 and 0.6 to 0.7 (two-sample *t* test: *t*s > 2.333, *p*s < 0.021). The Tri-factor model (*Eloc*) had better preserved global efficiency at steps of 0.2 and 0.6 to 0.8 (two-sample *t* test: *t*s > 2.630, *p*s < 0.010), and better preserved local efficiency at steps from 0.1 to 0.4 (two-sample *t* test: *t*s > 3.727, *p*s < 0.001). The Tri-factor model (*Q*) showed better preserved global efficiency at steps from 0.2 to 0.9 (two-sample *t* test: *t*s > 3.722, *p*s < 0.007), and better preserved local efficiency at steps from 0.1 to 0.8 (two-sample *t* test: *t*s > 2.333, *p*s < 0.001).

Under targeted attacks (Figure 5E), synthetic networks of the Dual-factor model (AUC of global efficiency = 0.213; AUC of local efficiency = 0.239) and the Tri-factor model (*Eloc*) (AUC of global efficiency = 0.084; AUC of local efficiency = 0.139) suffered more severe degeneration than the group-level empirical brain network (AUC of global efficiency = 0.236; AUC of local efficiency = 0.299), suggesting that networks of these two trade-off models were more vulnerable to attack on high-degree regions. Comparatively, the synthetic network of the Tri-factor model (*Q*) (AUC of global efficiency = 0.231; AUC of local efficiency = 0.291) was as resilient as the group-level empirical brain network. Hence, the representative synthetic network of the Tri-factor model (*Q*) could achieve similar robustness as the empirical brain network under both types of attacks. Combining the findings of the above sections, we could find that although a subtle difference remained when compared with the empirical ones, the Tri-factor model (*Q*) was the optimal trade-off model that underlaid the organization of the human brain network.

### Behavioral Relevance of Trade-Off Models

**Figure 6.**
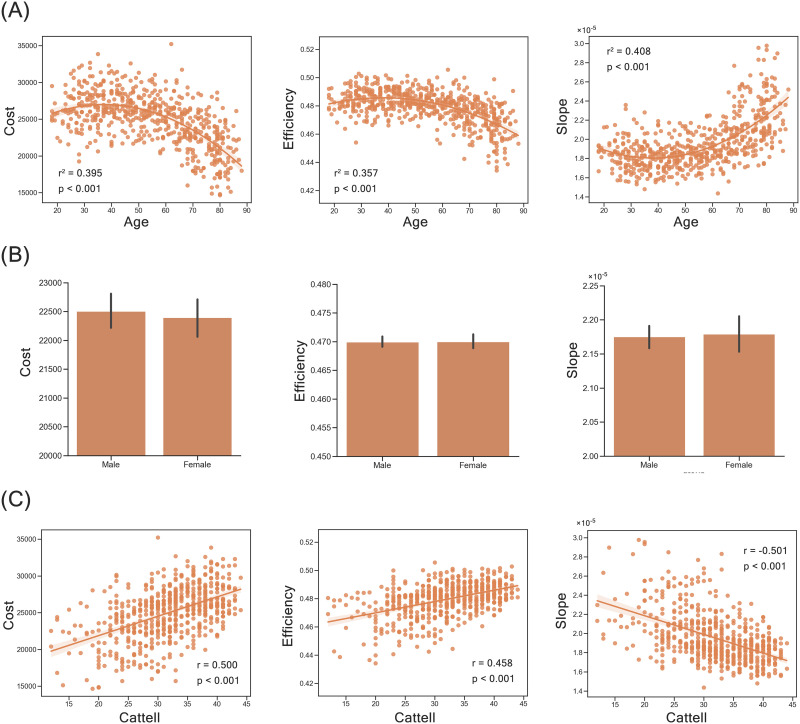
Behavioral results of the Tri-factor model (*Q*). (A) Scatterplot between age and morphospace indices (i.e., cost, efficiency, and slope). (B) Gender difference of morphospace indices (i.e., cost, efficiency, and slope). (C) Scatterplot between Cattell scores and morphospace indices (i.e., cost, efficiency, and slope). Slope is computed as efficiency/cost.

In addition to the recovery of the empirical brain structure, the optimal trade-off model, that is, the Tri-factor model (*Q*), could further capture individual human functions through individual variation of individual brain networks in the morphospace (Supporting Information Figure S2C). For demographic characteristics (i.e., age and gender), we observed that wiring cost (*r*^2^ = 0.395, *p* < 0.001) and efficiency index (*r*^2^ = 0.357, *p* < 0.001) of individual human brain networks showed inverted U-shaped trajectories with age, where wiring cost and communication efficiency of the human brain simultaneously increased before approximately 40 years old and decreased at older ages. Conversely, their slopes in morphospace (*r*^2^ = 0.408, *p* < 0.001) had a U-shaped trajectory with age (Figure 6A), indicating that the trade-off between cost and communication efficiency tended to promote communication efficiency before approximately 40 years and gradually deviated to minimizing cost at older ages. From the perspective of morphospace position (Supporting Information Figure S2C), it may suggest that individuals gradually move to the top right area as they age before middle age (around 40 years old), and then move back to the bottom left as they get older after middle age. As for gender difference, after controlling for the effect of age, we observed that the wiring cost (*p* = 0.007) and efficiency index (*p* = 0.008) were higher in the brain structural networks of male participants than in those of female participants (Figure 6B). The values of the slope metric (*p* = 0.032) were lower in the brain structural networks of male participants than in those of female participants (Figure 6B).

Besides the demographic characteristics, morphospace indices of the Tri-factor model (*Q*) could also capture individual behavioral performance (i.e., Cattell test). As shown in Figure 6C, wiring cost (*r* = 0.500, *p* < 0.001) and communication capacity (*r* = 0.458, *p* < 0.001) of individual human brain networks were positively correlated with the total scores of the Cattell test. Slopes of individual human brain networks were negatively correlated with the total scores of the Cattell test (*r* = −0.501, *p* < 0.001). Hence, individuals with efficient and costly brain networks had better fluid intelligence. Since the cost index is related to connection numbers, it may also mean that adding more connections in the human brain would be more beneficial. Moreover, we also found a negative correlation between age and Cattell scores (*r* = −0.660, *p* < 0.001; Supporting Information Figure S6). Combined with the above results, it may further suggest that cognitive decline (i.e., fluid intelligence here) in the aging progress is related to a simultaneous decrease of cost and efficiency in the structural human brain network. Besides the Tri-factor model (*Q*), we also analyzed the behavioral relevance of other trade-off models and found similar results (Supporting Information Table S2), which may be due to the identical cost index and slight difference in efficiency index in all models.

### Validation Results

To examine the reliability of trade-off models in recovering human brain networks, the main analyses of recovery rate and global topological properties were also repeated in the independent Cam-CAN sample. Similarly, empirical brain networks were dominated by the synthetic networks from all the trade-off models (Supporting Information Figure S2A–C). The Tri-factor model (*Q*) (*R* = 0.605 ± 0.014) showed a better recovery rate of connections than the Tri-factor model (*Eloc*) (*R* = 0.491 ± 0.060) and random networks (*R* = 0.400 ± 0.020) (two-sample *t* test: *t*s > 16.244, *p*s < 0.001), and a similar recovery rate as the Dual-factor model (*R* = 0.593 ± 0.058; two-sample *t* test: *t* = 1.881, *p* = 0.062; Supporting Information Figure S2D). Although the recovery rate is similar, the Tri-factor model (*Q*) outperformed the Dual-factor model in topological metrics of segregation capacity (e.g., clustering coefficient and modularity; two-sample *t* test: *t*s > 7.635, *p*s < 0.001) and small-worldness (two-sample *t* test: *t* = 52.538, *p* < 0.001; Supporting Information Figure S3 and Table S1). Topological dissimilarity analysis also revealed that the Tri-factor model (*Q*) had the lowest topological dissimilarity among the three models (two-sample *t* test: *ts* ≤ −33.666, *p*s < 0.001; Supporting Information Figure S4B). As in the main results, these findings consistently revealed the predominant performance of the Tri-factor model (*Q*) in recovering network structure of the empirical human brain and supported its optimality among the three trade-off models.

## DISCUSSION

In this study, we explored the optimal trade-off model that underlies organization of the human brain network using an MOEA approach and further examined its behavioral relevance. By modeling the trade-offs among fundamental factors (i.e., cost, integration, and segregation) as multiobjective optimization problems, we were able to generate synthetic networks under the optimal trade-off of corresponding models. These synthetic networks exhibited considerable performance in capturing the connectome topology of the empirical human brain network, including the overall connection pattern, network-level topological characteristics, and specific and nodal topological features. Through comparison among trade-off models, we found that the Tri-factor model (*Q*) had the best performance in segregated processing capacity, network robustness, nodal degree centrality, and recovery of middle- or long-distance connections, suggesting it as the optimal trade-off model of the human connectome. Finally, based on the morphospace of this optimal model, we further revealed that individual variation in the morphospace could not only capture performance in behavioral scores, but also reflect different correlation patterns in different behavioral domains.

### Trade-Off Among Cost, Segregation, and Integration

The human connectome wiring was widely hypothesized as the result of a trade-off between two competing factors: cost and efficiency (Bullmore & Sporns, 2012; Stiso & Bassett, 2018). Previous studies that examined this general principle mainly focus on the trade-off between cost and global efficiency (Y. Chen et al., 2013, 2017; Fornito et al., 2011; Ma et al., 2021), which neglected the effect of segregation (Sporns, 2013). In the current study, we further extended the cost-efficiency principle to consider the optimal trade-off among cost, integration, and segregation. Synthetic networks under an optimal trade-off among these factors showed high similarity with empirical brain networks in the edge pattern but fully dominated empirical brain networks in the morphospace. The implication of this suboptimality is twofold. First, it may suggest the existence of a trade-off, though a suboptimal trade-off among cost, integration, and segregation in the human brain network. This implication aligns with the previous notion that the connection placement of the human connectome may be at a suboptimal or near-optimal stage (Gollo et al., 2018). Second, the suboptimality implies that current trade-off models could not adequately capture all the features of the human brain network. Developmental constraints (Akarca et al., 2021; Nicosia, Vértes, Schafer, Latora, & Bullmore, 2013; Oldham et al., 2022) and other more specific constraints (e.g., cytoarchitectonic and genetic constraints; Arnatkeviciute et al., 2021) may be needed to complement the current framework.

Besides edge pattern, these synthetic networks were also able to reproduce several important features of the empirical human brain network that could not be explained by the original cost-efficiency model (Ma et al., 2021), including recovery of the segregated processing capacity (e.g., local efficiency and modularity), nodal degree centrality, and network robustness. The emergence of segregated properties was previously considered to be the result of cost minimization (Y. Chen et al., 2013, 2017; Sporns & Betzel, 2016), such as the sparse inter-module connections in modular structure. However, the segregated structure of the human brain network is also organized to achieve other important functions, including flexible and rapid specialized processing, and network robustness (Wig, 2017; Sporns & Betzel, 2016). Results of both the previous cost-efficiency model (Ma et al., 2021) and the current Dual-factor model also revealed that simply optimizing the trade-off between cost and global efficiency is inadequate to capture the segregation of the empirical brain networks. Rather, these models generated a more centralized network structure, where spatially central global hub regions owned dense connections to nearly all other regions. This structure could achieve efficient network communication with low cost, and did provide a good trade-off between cost and global efficiency. The trade-off models could capture the segregated properties of the empirical brain network only when segregated factors (e.g., modularity) were included as network constraints, suggesting the indispensable role of segregation in the wiring of the human brain network. Similar to our notion, previous research on small-worldness (Bassett & Bullmore, 2006, 2017; Liao et al., 2017), which describes high clustering and short average path length of networks, also suggested that the human connectome organization facilitated both integration and segregation of the brain (Deco et al., 2015). Besides small-worldness, many of the recapitulated network properties were previously attributed to other more specific constraints (Betzel et al., 2016; Markov et al., 2013; Roberts et al., 2016), such as the distance-dependent connection pattern (geometrical constraint) and nodal degree centrality (topological constraint). Hence, the trade-off among cost, integration, and segregation can be considered to be a more fundamental principle summarizing the effect of the above constraints. Current work based on the well-designed MOEA approach provided direct evidence on how this fundamental principle causally influences the brain’s network structure.

### Modularity as an Optimal Additional Factor of Trade-Off Models

Our study explored the effect of segregation in trade-off models from two aspects: local efficiency and modularity. Although both depict segregated capacity of the network (Cohen & D’Esposito, 2016), the trade-off model of modularity (i.e., Tri-factor model [*Q*]) obviously outperformed the model of local efficiency in most network features, such as overall recovery rate and robustness. This might be due to the adaptive properties of modular structure. First, modular structure of the brain network can not only support specialized neural processing, but also help conserve wiring cost (Y. Chen et al., 2013, 2017; Clune, Mouret, & Lipson, 2013). The spatially compact modules and their predominantly short within-module connections result in low global wiring cost of the network (Samu et al., 2014). Comparatively, local efficiency focuses on only the efficiency of local information transmission regardless of the global wiring cost (Latora & Marchiori, 2001). The cost conservation nature of modularity makes it a more suitable additional factor in the trade-off model. Second, the modular structure increases global robustness of network. On one hand, the dense within-modular connections and sparse between-modular connections of modular structure could confine the propagation of local perturbations within modules, which prevent or delay global distortion of the network (Sporns & Betzel, 2016; van den Heuvel & Sporns, 2019). On the other hand, as observed in our results, the pressure of modularity shifted the network from a single-core system to a multi-core system, where the spatial pattern of hubs became more distributed under the Tri-factor (*Q*) model. Accordingly, the robustness under targeted attack on high-degree regions was improved in the synthetic network. Together, the modular structure confers adaptive advantages to the network that were embedded in the evolution of the human brain rather than just segregation capacity. Moreover, it is worth noting that although local efficiency and modularity are two common segregation metrics, there are some other metrics with whichi to measure segregation of the network (e.g., system segregation, assortativity coefficient, and odds ratio; Bojanowski & Corten, 2014; Chan et al., 2014; Cohen & D’Esposito, 2016). Future work can incorporate more segregation metrics to further explore how the trade-off among cost, integration, and segregation shapes the human connectome.

### Degree Centrality and Long-Distance Connections of the Human Brain Network

Apart from segregation and robustness, the trade-off model of modularity also showed improved performance in recovering nodal degree centrality and long-distance connections. Both features are crucial to communication in brain networks. Degree centrality characterizes the numbers of direct connections owned by brain regions, reflecting their relative importance in network communication (van den Heuvel & Sporns, 2013; Zuo et al., 2012). The spatial distribution of degree centrality was considered to be a configuration of minimal wiring cost (Gollo et al., 2018). However, we could only find significant correlation in the Tri-factor model (*Q*), but not in other models that also accounted for the effect of minimizing cost, suggesting that nodal degree centrality is jointly determined by cost, integration, and modularity, rather than by cost alone. Moreover, despite the significant similarity, there remained a large variance of degree centrality that could not be explained by the Tri-factor model (*Q*). The spatial distribution of degree centrality might rely on not only the principle of fundamental factors (e.g., wiring cost and modularity), but also more specific nodal characteristics (e.g., cytoarchitectonic and genetic constraints). For example, recent work using a transcriptomic atlas data found a tight relationship between regional transcriptional activity and degree centrality of the structural (Arnatkeviciute et al., 2021) or functional (Zhu et al., 2021) human brain network. In future work, a model encompassing the trade-off principle and specific nodal information may better capture the spatial distribution of nodal degree.

Long-distance connections were costly components of the brain network that occupied a large amount of wiring cost. In return, they performed important roles in supporting direct communication between remote regions (Bassett & Bullmore, 2017; van den Heuvel & Sporns, 2011) and functional diversity of the network (Betzel & Bassett, 2018). In line with these suggestions, our model incorporating the trade-off among cost, integration, and modularity was able to reproduce a considerable portion of long-distance connections. However, compared with short-distance connections, the recovery rate of long-distance connections was relatively low. What are the roles of these remaining long-distance connections? One possible explanation is that these uncovered long-distance connections might serve as alternative nonoptimal pathways that support direct communication between areas when needed by specific task demands (Pappas et al., 2020). Under specific conditions (e.g., demanding cognitive tasks), brain network communication could reroute and take place on other suboptimal, potentially “expensive,” pathways (Avena-Koenigsberger et al., 2017).

### Shared and Fundamental Morphospace Underlies Human Functions

Based on the optimal trade-off model of the human brain network (i.e., the Tri-factor model [*Q*]), we proposed a morphospace that captured diverse demographic/behavioral characteristics of participants. In the past decade, mounting studies using network-based analyses have found a tight relationship between brain network topological characteristics and human behavior (Cohen & D’Esposito, 2016; Heitger et al., 2012; Park & Friston, 2013) or demographic characteristics (Cao et al., 2014; Ingalhalikar et al., 2014). Our morphospace, which represented a continuum of all possible human brain network configurations, was able to bridge the above brain-behavior relationship. On one hand, morphospace indices of individual brain networks had considerable correlation with both demographic characteristics and behavioral performance, showing a high sensitivity and generalizability in capturing different normal human functions. Findings of these correlations were also consistent with previous related results, such as U-shaped or inverted U-shaped trajectories of topological features (e.g., inter-/intra-module connectivity) along the life span (Cao et al., 2014; Luo et al., 2020; Zuo et al., 2017) and positive correlation between network properties (e.g., global efficiency) and intelligence (Fischer et al., 2014; Li et al., 2009). Our morphospace synthesized all these relationships from a more general and fundamental perspective. On the other hand, individual characteristics from different domains showed different correlation patterns with the three morphospace indices (e.g., inverted U-shaped trajectories with age, and negative linear correlation with Cattell scores), suggesting a specificity of the trade-off morphospace. Hence, variation in the morphospace could not only capture common variance of individual characteristics, but also reflect unique information of different characteristics. With the above advantages, the Tri-factor model (*Q*) could serve as a fundamental and shared morphospace that underlies various human functions of participants. Although the current study focused on healthy adults, the present morphospace might be also be able to capture diagnostic and symptom-specific abnormality of individuals through aberrant reconfiguration of the brain network structure along the morphospace dimensions (van den Heuvel & Sporns, 2019). This notion aligns with recent initiatives on neurobiological dimension-based diagnostic approaches (Insel et al., 2010; Kebets et al., 2021). Future work with participants from diverse diagnostic categories could adopt the present morphospace to verify this hypothesis.

### Computational Models in Recapitulating the Organization of the Human Connectome

We note that our MOEA approach is similar to several existing computational models that try to recapitulate the organization of the empirical brain network. For instance, the generative model (Akarca et al., 2021; Betzel et al., 2016; Vértes et al., 2012; Zhang et al., 2021) generates synthetic networks with the probability determined by nodal geometric and topological features, which can clarify contribution of these features and reveal potential factors driving the formation of connections. Another related method is the random network model (Gollo et al., 2018; Roberts et al., 2016; Samu et al., 2014), which randomizes network connections while preserving certain network properties (e.g., geometry) and examines similarity with the empirical brain network. Although similar, the MOEA approach has two unique features. First, the MOEA model is more theory-driven. Synthetic networks are constructed based on the cost-efficiency trade-off principle (Bullmore & Sporns, 2012) but not on explicit guiding information of empirical brain networks (e.g., empirical local topology as in the generative model), which allows direct examination of the effect of a specific trade-off model and further reveals the fundamental force (e.g., evolutionary force of cost and efficiency) underlying brain development. Second, the MOEA approach is able to simultaneously optimize multiple competing factors. This advantage makes MOEA a suitable approach for solving trade-off problems, not only cost-efficiency trade-off problems, but also other problems (e.g., the trade-off between within- and between-modular connection density in modular partition; Gu et al., 2022; Lin et al., 2018).

### Additional Considerations

Despite the novel findings, some limitations should be taken into consideration. First, our study only investigated the trade-off between cost and efficiency of structural networks. The efficiency of functional networks, which might be more directly related to communication efficiency of the human brain (Laughlin & Sejnowski, 2003), was not considered. Whether efficiency of functional networks is another driving factor of the formation of the human connectome remains an open question. Future work incorporating multimodal neuroimaging data (e.g., PET and fMRI) and functional network prediction models (e.g., communication models and biophysical models; Avena-Koenigsberger, Misic, & Sporns, 2018; Deco et al., 2014; Demirtaş et al., 2019; Suárez et al., 2020) might be able to more accurately infer how synthetic networks under trade-off models support human brain functions. Second, to approximate the neural wiring cost, we computed the sum of the Euclidean distance between connected brain areas. Since network connections constructed in the simulation process might not correspond to actual connections in the empirical human brain network, which makes it hard to evaluate the actual fiber length, Euclidean distance is a suitable proxy of connection length (Y. Chen et al., 2013; Ma et al., 2021). However, Euclidean distance between brain regions, especially interhemispheric region pairs, does not strictly follow the exact fiber length, which might introduce bias in cost evaluation. Moreover, besides connection length, properties like fiber number and connection diameter also play a central role in wiring cost (Bullmore & Sporns, 2012). Directly quantifying these properties in MOEA simulation is often difficult. Therefore, in future work, a computational model that could more precisely estimate connection cost is needed. Third, our examination of trade-off models was mainly based on diffusion MRI data of the empirical human brain and did not link to other empirical neurobiological phenomena. In the future, combining multimodal imaging data and linking the neurobiological measures (e.g., T1/T2 ratio; Oldham et al., 2022) with synthetic networks could provide converging evidence of the trade-off principle in the human connectome. Fourth, with the consideration of reliability and high computational load of the MOEA approach, we used the classical and reliable AAL atlas for node definition in the current study. However, this nodal parcellation is relatively sparse and a different choice of nodal parcellation may induce a different topological outcome (Zalesky et al., 2010). A parcellation with higher resolution would be needed to further validate our findings in future studies.

## ETHICS STATEMENT

This study was approved by the Institutional Review Board in the Department of Psychology of Sun Yat-sen University, and all participants provided informed consent before the experiment.

## DATA AND CODE AVAILABILITY

The newly acquired SCNU dataset for the present study is available at an open platform (https://osf.io/ebtks/?view_only=7f4a89f06c464da7b32253c7f3bd0f3d; Zhengjia, 2022). Data of the Cam-CAN dataset are collected from a published database of the Cambridge Centre for Ageing and Neuroscience. The raw data of the Cam-CAN dataset are available at https://camcan-archive.mrc-cbu.cam.ac.uk/dataaccess/. The toolboxes and third-party code we used are all stated and cited appropriately, and relevant links of code are also provided in the Materials and Methods section. Pseudocode of our implemented NSGA-II algorithm can be found in Algorithm S1 in the Supporting Information.

## SUPPORTING INFORMATION

Supporting information for this article is available at https://doi.org/10.1162/netn_a_00291.

## AUTHOR CONTRIBUTIONS

Junji Ma: Formal analysis; Methodology; Validation; Visualization; Writing – original draft; Writing – review & editing. Xitian Chen: Formal analysis; Visualization; Writing – review & editing. Yue Gu: Methodology; Validation; Writing – review & editing. Liangfang Li: Methodology; Validation; Writing – review & editing. Ying Lin: Conceptualization; Methodology; Supervision; Writing – review & editing. Zhengjia Dai: Conceptualization; Funding acquisition; Methodology; Supervision; Writing – review & editing.

## FUNDING INFORMATION

Zhengjia Dai, STI2030-Major Projects of China, Award ID: 2022ZD0213300. Ying Lin, National Natural Science Foundation of China (NSFC), Award ID: 61772569. Zhengjia Dai, Guangdong Basic and Applied Basic Research Foundation (https://dx.doi.org/10.13039/501100021171), Award ID: 2022A1515012005. Zhengjia Dai, Guangdong Basic and Applied Basic Research Foundation (https://dx.doi.org/10.13039/501100021171), Award ID: 2019A1515012148. Zhengjia Dai, Open Research Fund of the State Key Laboratory of Cognitive Neuroscience and Learning, Award ID: CNLYB2001. Zhengjia Dai, Fundamental Research Funds for the Central Universities (https://dx.doi.org/10.13039/501100012226), Award ID: 19wkzd20.

## TECHNICAL TERMS

Human connectome: Structural human brain network with brain regions as nodes and white matter connections as edges.
Cost-efficiency trade-off: An optimal trade-off between wiring cost and communication efficiency in the human brain network.
Integration: Global neural communication among all regions of the brain network.
Segregation: Local and specialized neural processing within clusters of brain regions.
Multiobjective evolutionary algorithm: One type of search algorithm that could simultaneously optimize multiple objectives by imitating natural evolutionary processes.
Non-dominated relationship: Two solutions are non-dominated over each other if neither of them can have an advantage over the other one on all the optimization objectives of the given multiobjective problem.
Non-dominated solution: A solution is non-dominated if it is feasible and cannot be improved on any optimization objective without worsening the other objectives.
Pareto front: A front composed of non-dominated solutions to a multiobjective problem in the morphospace.
Morphospace: The objective space of a multiobjective problem, with each dimension defined as an objective of the corresponding problem.

## Supporting Information

https://osf.io/ebtks/?view_only=7f4a89f06c464da7b32253c7f3bd0f3d
