# Supplementary material for: Trade-offs among cost, integration, and segregation in the human connectome
Source: Netw Neurosci. 2023 Jun 30;7(2):604–31. doi: 10.1162/netn_a_00291 (PMC10312266; doi:10.1162/netn_a_00291)
Supplement: Supplementary file 1 [file netn-7-2-604-s001.pdf]

Ma, J., Chen, X., Gu, Y, Li, L., Lin, Y. & Dai, Z. (2022). Supporting information for "Trade-offs among cost, integration, and segregation in the human connectome." *Network Neuroscience*. Advance publication.
https://doi.org/10.1162/netn_a_00291

Supplementary Materials

Algorithm S1: NSGA-II

Input:

- N: the number of nodes in network
- gen_{max}: the maximum number of generations
- gen_{stag}: the maximum number of consecutive stagnation
- P: the population size

Output:

- S: the population of synthetic networks

// Generate the synthetic networks by simultaneously minimizing cost and maximizing
// efficiency
// Population initialization
FOR $i = 1$ **TO** P
    Randomly assign 1 or 0 values to each entry in solution S_i;
    Evaluate objectives of solution S_i;
END
// Evolution process
$stag \leftarrow 0$; $gen \leftarrow 1$;
WHILE $gen < gen_{max}$ and $stag < gen_{stag}$
    $Sp \leftarrow$ selection(S_{new});   // Select high-quality parental population Sp
    $O \leftarrow$ crossover(Sp);   // Perform crossover to generate offspring O
    Evaluate_objectives(O);
    $X \leftarrow$ mutation($Sp \cup O$);   // Perform mutation to generate new population X
    Evaluate_objectives(X);
    $S_{new} \leftarrow$ nondominated_sorting(X);   // Select new population S_{new}
    // Check consecutive stagnation
    $count \leftarrow$ length($S \cap S_{new}$);
    $diff \leftarrow$ max(|objectives of S – objectives of S_{new}|)/mean(objectives of S);
    IF $count >= 0.95*P$ **OR** $diff < 0.001$
        $stag \leftarrow stag + 1$;
    ELSE
        $stag \leftarrow 0$;
    END
    $S \leftarrow S_{new}$;
    $gen \leftarrow gen + 1$;
END
RETURN S

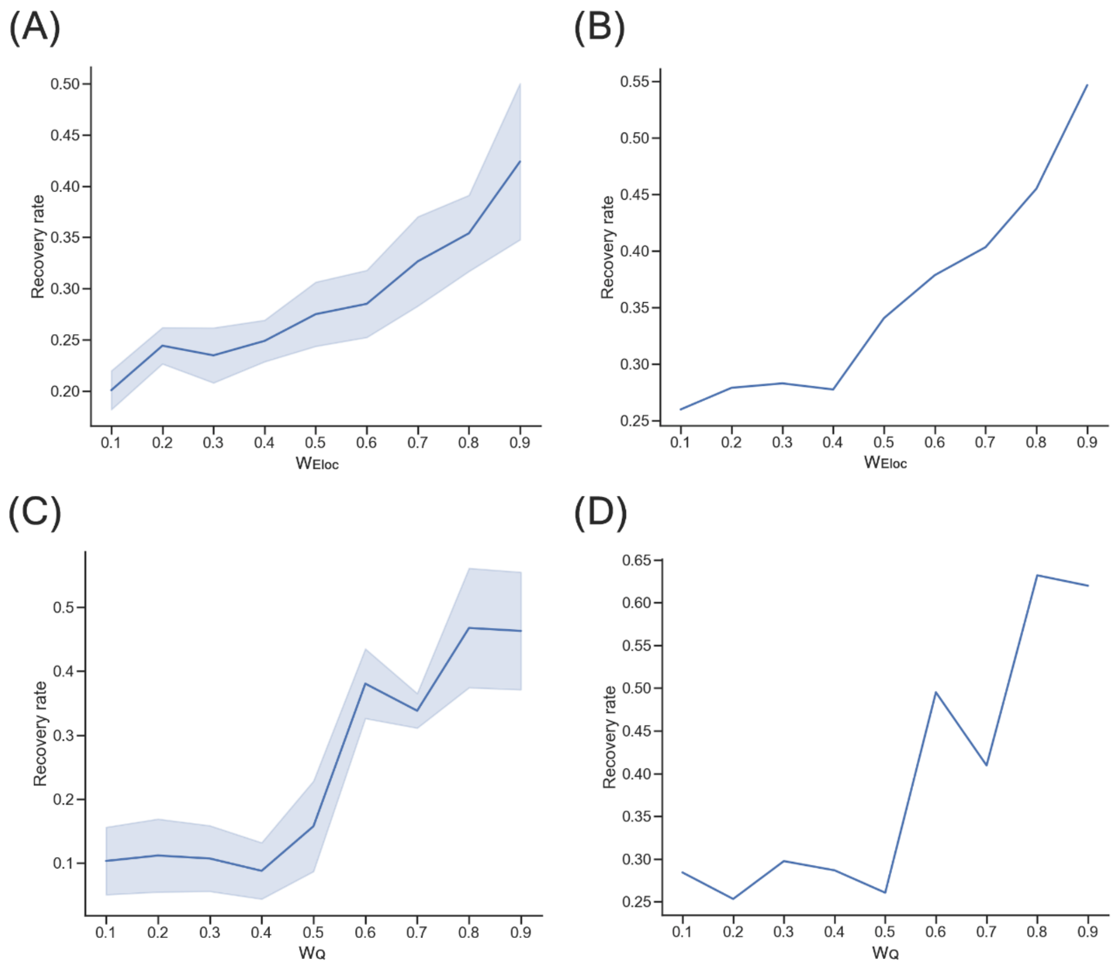

Figure S1. Recovery rates of synthetic networks under different values of w_{Eloc} or w_Q. (A) Recovery rates of synthetic networks under values of w_{Eloc}, and the shadow of lines indicates the range of one standard deviation. (B) Maximum recovery rates of synthetic networks under values of w_{Eloc}. (C) Recovery rates of synthetic networks under values of w_Q, and the shadow of lines indicates the range of one standard deviation. (D) Maximum recovery rates of synthetic networks under values of w_Q.

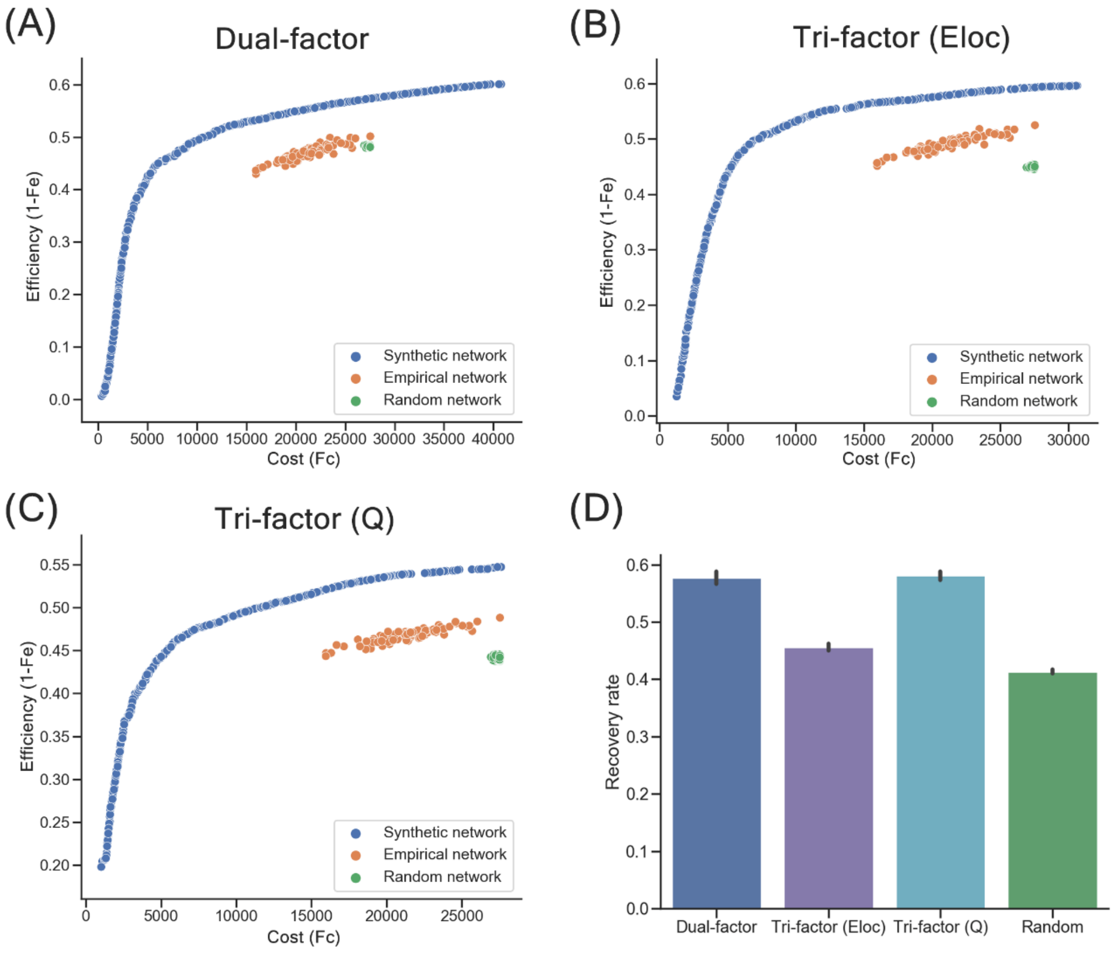

Figure S2. Synthetic networks of trade-off models for validation analyses. (A) Distribution of synthetic networks (blue points), empirical brain networks (Cam-CAN dataset; orange points), random networks (green points) in morphospace of the Dual-factor model. (B) Distribution of networks in morphospace of the Tri-factor model (Eloc). (C) Distribution of networks in morphospace of the Tri-factor model (Q). Notably, the values of the F_e objective were transformed by one minus F_e (i.e., efficiency index). (D) Recovery rates of synthetic networks and random networks. Recovery rates were significantly different between network groups (ps < 0.001), except for comparison between Dual factor model and Tri-factor model (Q) (p = 0.062).

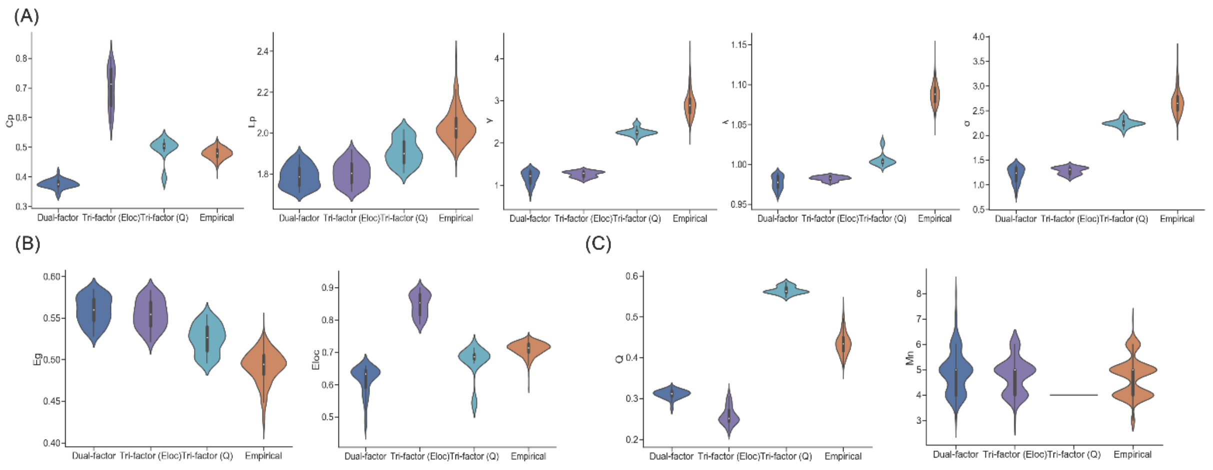

Figure S3. Network-level topological characteristics of synthetic networks and empirical brain networks from Cam-CAN dataset. (A) Clustering coefficient (Cp), characteristic path length (Lp), normalized clustering coefficient (γ), normalized characteristic path length (λ), and small-worldness (σ) of synthetic networks and empirical brain networks. Small-world metrics were significantly different between network groups (ps < 0.001), except for comparison between module number of Dual factor model and Tri-factor model (Eloc) (p = 0.066). (B) Global efficiency (Eg) and local efficiency (El) of synthetic networks and empirical brain networks. Efficiency metrics were significantly different between network groups (ps < 0.001), except for comparison between module number of Dual factor model and Tri-factor model (Eloc) (p = 0.069). (C) Modularity (Q) and module number (Mn) of synthetic networks and empirical brain networks. Modular metrics were significantly different between network groups (ps < 0.001), except for comparison between module number of Dual factor model and Tri-factor model (Eloc) (p = 0.233). Note, the synthetic networks are the networks whose cost values are distributed in the range of the Cam-CAN dataset.

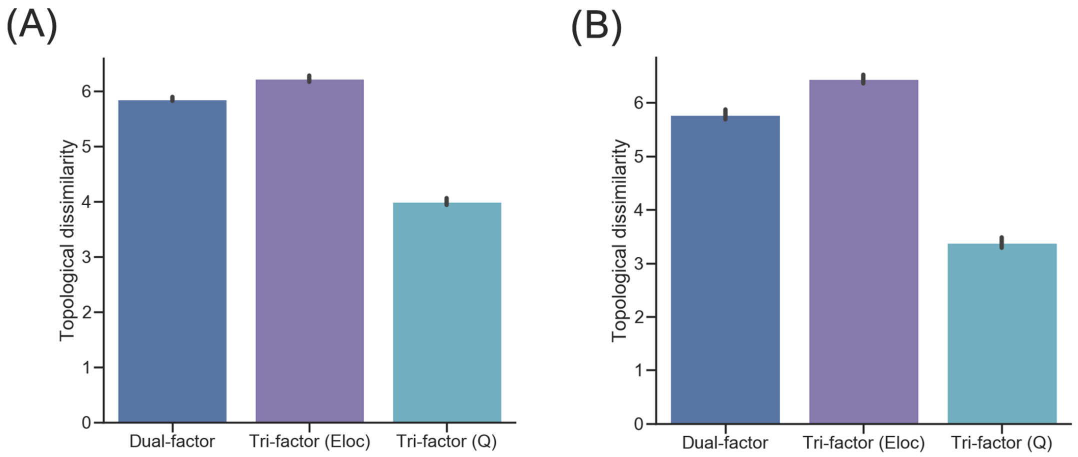

Figure S4. Topological dissimilarity results of trade-off models. (A) Topological dissimilarity to the empirical brain networks in SCNU sample. (B) Topological dissimilarity to the empirical brain networks in Cam-CAN sample.

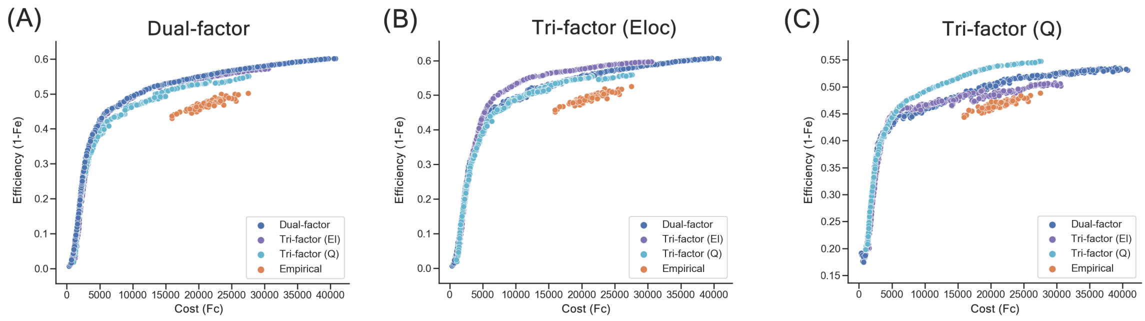

Figure S5. Distribution of synthetic networks under three trade-off models and empirical brain networks in morphospace. (A) Distribution of networks in morphospace of the Dual-factor model. (B) Distribution of networks in morphospace of the Tri-factor model (Eloc). (C) Distribution of networks in morphospace of the Tri-factor model (Q).

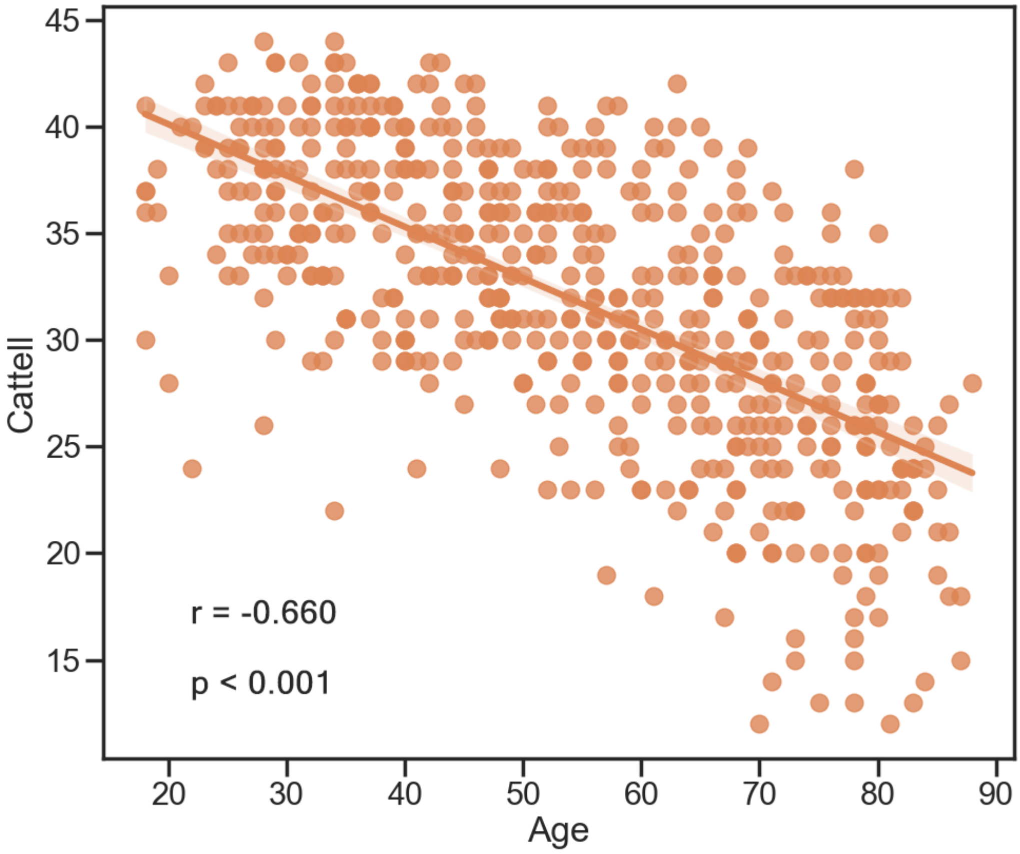

Figure S6. Scatter plot between age and Cattell scores in Cam-CAN sample.

Table S1 Network-level graph metrics

Graph metrics	Dual-factor		Tri-factor (Eloc)		Tri-factor (Q)		Empirical	
	value	t	value	t	value	t	value	t
Cp	0.376 ± 0.015	136.312***	0.700 ± 0.068	82.788***	0.492 ± 0.039	90.700***	0.479 ± 0.09	183.339***
Lp	1.790 ± 0.053	-35.729***	1.805 ± 0.056	-30.794***	1.908 ± 0.063	-11.118***	2.038 ± 0.086	6.854***
γ	1.175 ± 0.164	-	1.259 ± 0.071	-	2.259 ± 0.094	-	2.913 ± 0.293	-
λ	0.977 ± 0.008	-	0.983 ± 0.003	-	1.006 ± 0.009	-	1.088 ± 0.014	-
σ	1.201 ± 0.160	-	1.281 ± 0.074	-	2.244 ± 0.078	-	2.674 ± 0.240	-
Eg	0.559 ± 0.016	32.799***	0.555 ± 0.017	28.475***	0.525 ± 0.017	11.103***	0.492 ± 0.020	-6.932***
Eloc	0.616 ± 0.044	83.145***	0.847 ± 0.034	156.220***	0.670 ± 0.050	83.815***	0.711 ± 0.021	224.729***
Q	0.311 ± 0.011	38.411***	0.258 ± 0.022	-0.175n.s.	0.565 ± 0.009	232.775***	0.621 ± 0.027	64.043***
Mn	4.908 ± 0.826	-12.803***	4.769 ± 0.682	-14.260***	4.000 ± 0	-26.110***	4.701 ± 0.755	-20.484***

Notes: Cp, clustering coefficient; Lp, characteristic shortest path length; γ, normalized clustering coefficient; λ, normalized characteristic shortest path length; σ, small-worldness; Eg, global efficiency; Eloc, local efficiency; Q, modularity; Mn, module number; value, Mean ± SD of the metric score; t, t scores of two-sample t test between metric scores of corresponding network group and random networks; Since computation of γ, λ, and σ have been normalized with random networks, we do not provide t scores for these metrics; *** $p < 0.001$, n.s. $p > 0.05$

Table S2 Behavioral results of trade-off models

	Dual-factor			Tri-factor (Eloc)			Tri-factor (Q)		
	Cost	Efficiency	Slope	Cost	Efficiency	Slope	Cost	Efficiency	Slope
Age (r^2)	0.395^{***}	0.341^{***}	0.418^{***}	0.395^{***}	0.355^{***}	0.414^{***}	0.395^{***}	0.357^{***}	0.408^{***}
Gender (diff)	522.379^{**}	0.003^{**}	-0.028^{*}	522.379^{**}	0.003^{**}	-0.029^{*}	522.379^{**}	0.002^{**}	-0.011^{*}
Cattell (r)	0.500^{***}	0.453^{***}	0.507^{***}	0.500^{***}	0.462^{***}	-0.505^{***}	0.500^{***}	0.458^{***}	0.501^{***}

Notes: diff, gender difference (i.e., male - female) between group mean morphospace indices; * $p < 0.05$, ** $p < 0.01$, *** $p < 0.001$

Methods

Topological Characteristic of the Networks

During evaluation of objective functions and subsequent topological analyses, we calculated the graphical metrics at the global network level. Calculation of the graphical metrics was all performed using Graph Theoretical Network Analysis toolbox (GRETNA; Wang et al., 2015) and Brain Connectivity Toolbox (BCT; Rubinov & Sporns, 2010). Following is the detailed definition of graph metrics:

- Clustering coefficient (Cp) of network:

$$\mathrm{Cp} = \frac{1}{N}\sum_{i \in V}\left(\frac{E_i}{\frac{1}{2}k_i(k_i - 1)}\right)$$

Where N is the total number of nodes, here $N = 90$; V is the set of all nodes; E_i is the number of edges among neighbors of node i; k_i is degree centrality of node i. Normalized clustering coefficient γ is calculated as $\mathrm{Cp}/\mathrm{Cp}_{\mathrm{rand}}$, where $\mathrm{Cp}_{\mathrm{rand}}$ is the mean Cp of 100 topological random networks with preserved degree distribution. Both metrics evaluates the connection density among neighboring regions (Watts & Strogatz, 1998).

- Characteristic path length (Lp) of network:

$$\mathrm{Lp} = \frac{1}{N(N-1)}\sum_{i,j \in V, i \neq j} l_{ij}$$

Where N is the total number of nodes, here $N = 90$; V is the set of all nodes; l_{ij} is the length of shortest path between node i and j. Normalized characteristic path length λ is calculated as $\mathrm{Lp}/\mathrm{Lp}_{\mathrm{rand}}$, where $\mathrm{Lp}_{\mathrm{rand}}$ is the mean Lp of 100 topological random networks with preserved degree distribution. Both metrics evaluates the topological distance (i.e., path length) between region pairs (Watts & Strogatz, 1998).

- Small-worldness (σ) of nework:

$$\sigma = \frac{\gamma}{\lambda}$$

Where γ is normalized clustering coefficient and λ is normalized normalized characteristic path length. This metric evaluates the balance between integration and segregation of network (Watts and Strogatz, 1998).

- Global efficiency (Eg) of network:

$$\text{Eg} = \frac{1}{N}\sum_{i=1}^{N}\frac{\sum_{j=1}^{N} 1/l_{ij}}{N-1}, \qquad i \neq j = 1,2,3 \dots\dots N$$

Where N is the total number of nodes, here $N = 90$; l_{ij} is the length of shortest path between node i and j. This metric evaluates efficiency between all regions in the network (Latora and Marchiori, 2001).

- Local efficiency (Eloc) of network:

$$\text{Eloc} = \frac{1}{N}\sum_{i=1}^{N} Eg(G_i), \qquad i \neq j = 1,2,3 \dots\dots N$$

Where N is the total number of nodes, here $N = 90$; G_i is the local subgraph of node i that contains only its direct neighbors and $Eg(G_i)$ is the global efficiency of subgraph G_i. This metric evaluates the mean efficiency of regional subnetwork that is directly connected to target regions (Latora & Marchiori, 2001).

- Modularity (Q) of network:

$$\text{Q} = \frac{1}{u}\sum_{i,j\in V}(a_{ij} - \frac{k_i k_j}{u})\delta_{m_i,m_j}, \qquad i \neq j = 1,2,3 \dots\dots N$$

Where V is the set of all nodes; a_{ij} is the value of edge in binary network A; k_i is the degree centrality of node i; $u = \sum_{i\in V} k_i$ is the total number of edges in the network; m_i is the modular assignment of node i, and $\delta_{m_i,m_j} = 1$ if $m_i = m_j$, and 0 otherwise. This metrics

evaluates the quality of network modular structure (Newman, 2006), which is obtained from Louvain community detection algorithm (Blondel et al., 2008). Module number (Mn) is calculated as the number of modules in modular partition.

Specific Features of the Representative Synthetic Network

In addition to the global topological characteristics, we further investigate other more specific network properties (e.g., nodal degree centrality) that were also crucial for network function (e.g., integration) of human brain (Aerts et al., 2016; Sporns, 2013; van den Heuvel & Sporns, 2013). Here, we examined three aspects of network properties, which were modular structure, nodal degree centrality, and robustness of network.

Modular Structure. Modularization refers to a network structure with dense connections within clusters of nodes and sparse connections between clusters, which promotes segregated processing of network (Meunier et al., 2009). The modular structure is crucial for segregated processing of human brain network (Sporns & Betzel, 2016). To explore the modular partition of different trade-off models and compare their similarity with the empirical network, we applied the Louvain community detection algorithm (Blondel et al., 2008) on the three representative synthetic networks and the group-level empirical brain network to obtain their modular partitions respectively. Similarity of modular partition were then compared using the Kappa index between partitions of synthetic networks and empirical network. Kappa index is a measure to access the performance of classifier by accounting the actual percentage of correct classification compared to the expected probability by chance.

Degree Centrality and Hubs. The existence of hub regions that own a relatively large number

of connections is an important feature of human brain network that support the integration of information (Sporns, 2013). Appropriate arrangement of degree centrality of brain regions could not only minimize wiring cost but also promote the global integration of network (Gollo et al., 2018). In the current analysis, we first computed the nodal degree centrality (i.e., number of connections per node; Zuo et al., 2012) in the representative synthetic networks and the group-level empirical brain network, respectively. Pearson correlation was computed between the two nodal degree centrality vectors to evaluate their similarity in the degree distributions of the representative synthetic networks and the group-level empirical brain network. Then, the top 20% highest degree regions were identified as hubs (Collin et al., 2016; van den Heuvel et al., 2010; Wang et al., 2018), and their spatial locations were compared.

Robustness of Network. Empirical human brain network can be remarkably robust to damage (Aerts et al., 2016; Kaiser et al., 2007). This robustness is an important property that allows brain network to maintain normal functions after adverse perturbation (i.e., lesion). Hence, we wanted to investigate whether the synthetic networks constructed under different models could also recover the remarkable robustness as the empirical one. We performed computational attacks on the representative synthetic and empirical brain networks respectively. The degree of network degeneration was evaluated in terms of global efficiency and local efficiency. Two types of computational attacks were conducted: random attacks and targeted attacks. For the random attacks, nodes were removed from the network in a random order step by step. The procedure was repeated for 100 times to avoid bias from randomness and the 100 attacked networks were reported for analyses. For the targeted attacks, the nodes

were deleted in descending order of degree centrality step by step. For both types of attacks, the step length was set the same as 10% of nodes in the entire network.

References

Aerts, H., Fias, W., Caeyenberghs, K., & Marinazzo, D. 2016. Brain networks under attack: robustness properties and the impact of lesions. Brain 139(12), 3063-3083.

Blondel, V.D., Guillaume, J.-L., Lambiotte, R., Lefebvre, E., 2008. Fast unfolding of communities in large networks. Journal of statistical mechanics: theory experiment 2008, P10008.

Collin, G., van den Heuvel, M. P., Abramovic, L., Vreeker, A., de Reus, M. A., van Haren, N. E., ... & Kahn, R. S., 2016. Brain network analysis reveals affected connectome structure in bipolar I disorder. Human Brain Mapping, 37(1), 122-134.

Gollo, L.L., Roberts, J.A., Cropley, V.L., Di Biase, M.A., Pantelis, C., Zalesky, A., Breakspear, M., 2018. Fragility and volatility of structural hubs in the human connectome. Nature Neuroscience 21, 1107-1116.

Kaiser, M., Martin, R., Andras, P., & Young, M. P. (2007). Simulation of robustness against lesions of cortical networks. European Journal of Neuroscience 25(10), 3185-3192.

Latora, V., Marchiori, M., 2001. Efficient behavior of small-world networks. Phys Rev Lett 87, 198701.

Meunier, D., Lambiotte, R., Fornito, A., Ersche, K., Bullmore, E.T., 2009. Hierarchical modularity in human brain functional networks. Frontiers in neuroinformatics 3, 37.

Newman, M.E.J., 2006. Modularity and community structure in networks. Proc Natl Acad Sci. U. S. A. 103, 8577–8582.

Rubinov, M., Sporns, O., 2010. Complex network measures of brain connectivity: uses and interpretations. Neuroimage 52, 1059-1069.

Sporns, O., & Betzel, R. F., 2016. Modular brain networks. Annual review of psychology 67, 613-640.

Sporns, O. 2013. Network attributes for segregation and integration in the human brain. Current Opinion in Neurobiology 23(2), 162-171.

van den Heuvel, M. P., Mandl, R. C., Stam, C. J., Kahn, R. S., & Pol, H. E. H., 2010. Aberrant frontal and temporal complex network structure in schizophrenia: a graph theoretical analysis. Journal of Neuroscience 30(47), 15915-15926.

van den Heuvel, M.P., Sporns, O., 2013. Network hubs in the human brain. Trends in Cognitive Sciences 17, 683-696.

Wang, J., Wang, X., Xia, M., Liao, X., Evans, A., He, Y., 2015. GRETNA: a graph theoretical network analysis toolbox for imaging connectomics. Front Hum Neurosci 9, 386.

Wang, X., Lin, Q., Xia, M., & He, Y., 2018. Differentially categorized structural brain hubs are involved in different microstructural, functional, and cognitive characteristics and contribute to individual identification. Human Brain Mapping 39(4), 1647-1663.

Watts, D.J., Strogatz, S.H., 1998. Collective dynamics of 'small-world' networks. Nature 393, 440-442.

Zuo, X.-N., Ehmke, R., Mennes, M., Imperati, D., Castellanos, F.X., Sporns, O., Milham, M.P., 2012. Network centrality in the human functional connectome. Cerebral Cortex 22, 1862-1875.
